# Tuning the Acceptor Unit of Push–Pull Porphyrazines for Dye-Sensitized Solar Cells

**DOI:** 10.3390/molecules26082129

**Published:** 2021-04-07

**Authors:** Diana-Paola Medina, Javier Fernández-Ariza, Maxence Urbani, Frédéric Sauvage, Tomás Torres, M. Salomé Rodríguez-Morgade

**Affiliations:** 1 Departamento de Química Orgánica, Universidad Autónoma de Madrid, Cantoblanco, 28049 Madrid, Spain; dpmedina13@gmail.com (D.-P.M.); javier.f.ariza@gmail.com (J.F.-A.); 2 Instituto Madrileño de Estudios Avanzados (IMDEA)-Nanociencia, c/ Faraday, 9, Cantoblanco, 28049 Madrid, Spain; maxence.urbani@uam.es; 3 Laboratoire de Réactivité et Chimie des Solides, Université de Picardie Jules Verne, CNRS UMR7314, Hub de L’énergie, 15 rue Baudelocque, 80039 Amiens, France; frederic.sauvage@u-picardie.fr

**Keywords:** porphyrazines, push–pull systems, amphiphilic systems, dye-sensitized solar cells, photovoltaics

## Abstract

A family of four push–pull porphyrazines of A_3_B type, where each unit A contains two peripheral propyl chains and the unit B is endowed with a carboxylic acid, were prepared. The carboxylic acid was attached to the β-position of the pyrrolic unit, either directly (Pz **10**), or through cyanovinyl (Pz **11**) and phenyl (Pz **7**) groups. The fourth Pz (**14**) consisted in a pyrazinoporphyrazine wherein the dinitrogenated heterocycle provided intrinsic donor–acceptor character to the macrocycle and contained a carboxyphenyl substituent. The direct attachment of the carboxylic acid functions and their linkers to the porphyrazine core produces stronger perturbation on the electronic properties of the macrocycle, with respect to their connection through fused benzene or pyrazine rings in **TT112** and **14**, respectively. The HOMO and LUMO energies of the Pzs, which were estimated with DFT calculations, show little variation within the series, except upon introduction of the cyanovinyl spacer, which produces a decrease in both frontier orbital energetic levels. This effective interaction of cyanovinyl substitution with the macrocycle is also evidenced in UV/Vis spectroscopy, where a large splitting of the Q-band indicates strong desymmetrization of the Pz. The performance of the four Pzs as photosensitizers in DSSCs were also investigated.

## 1. Introduction

Owing to the vast number of studies performed on porphyrins (Ps) and phthalocyanines (Pcs) together with their applications in manifold fields, these porphyrinoids constitute the two main representatives of tetrapyrrolic macrocycles. Porphyrazines [1,2,3,4,5] (Pzs, tetraazaporphyrins) consist of tetraaza-analogues of porphyrins that share many of their important attributes, such as their aromaticity, their 18 π-electron aromatic circuit and their ability to efficiently absorb visible light. Although being strict, phthalocyanines consist of porphyrazines containing four fused benzene rings, the chemistry and applications of the former are so extensive [6] that they are considered a group apart, and the name of “porphyrazine” is usually reserved to tetraazaporphyrins that do not contain such fused benzene rings. Both Pcs and Pzs display absorption envelopes dominated by the so-called B and Q-bands. But while the bare ZnPz shows its Q-band at 580 nm [1], the extended conjugation of ZnPc produces the corresponding absorption at 670 nm [7,8]. However, Pzs show excellent optical and redox tunability, which can be achieved by selecting a specific central cation and/or attaching appropriate functional groups to the *β*-positions of the pyrrole rings. By doing so, peripheral substituents strongly interact with the macrocyclic core, to an extent that is not possible in Pcs, where the substituents are not connected directly, but through benzene rings. For example, octaamino-ZnPzs show their Q-band at 718 nm in addition to an intense (70% of Q-band intensity) CT band at 546 nm, arising from n→π* transitions, enabled by strong π-donation from the nitrogen lone pair to the Pz macrocycle [9]. Similarly, octaamino-ZnPcs show red-shifted Q-bands at 732 nm and CT bands at 522 nm (15% of Q-band intensity) [10]. Clearly, the absorption properties of the Pz macrocycle are far more disturbed upon peripheral substitution with amines than those of Pcs. Peripheral functionalization also impacts the redox properties of Pzs in higher extent than in Pcs. Thus, in octaamino-Pzs, the MgPz/MgPz^+^ and MgPz/MgPz^−^ couples appear at −0.27 V and −1.61 V, respectively [11], whereas the MgPc/MgPc^+^ and MgPc/MgPc^−^ couples of the corresponding octaamino-Pcs are detected at +0.09 and −1.36 V, respectively [10].

Other types of porphyrazines contain annulated heterocycles at their pyrrole rings, the nature of which determines the electronic character of the macrocycle [12,13,14,15]. Thus, for example, tetrapyrazinoporphyrazines [16,17], containing eight extra nitrogen atoms related to phthalocyanines, display their Q-band at around 635 nm [18] and are stronger electron-deficient chromophores, being around 440 mV easier to reduce than the corresponding Pcs [19].

Despite their above-mentioned possibilities, porphyrazines remain quite unexplored in comparison with Pcs and Ps, perhaps because the impact produced in the macrocycle by their peripheral substitution could turn out to be associated to less stability in some cases [20]. Still, even this aspect can be used as an added value for some applications [21,22]. 

**Figure 1:**
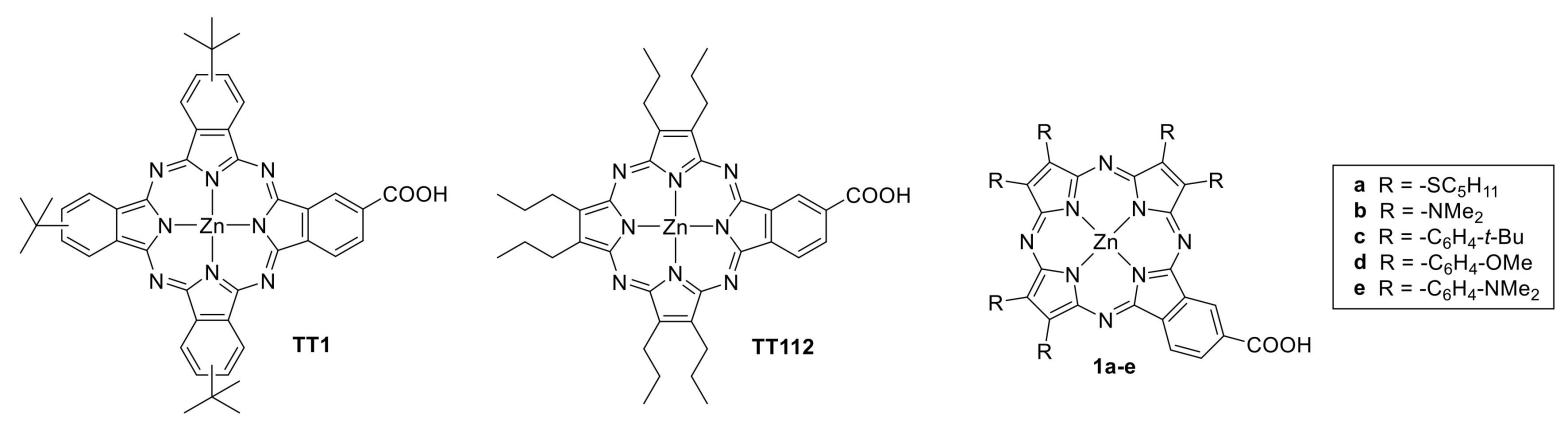
Chemical structures of Pc **TT1**, **TT112**, and Pzs **1a-e**.

Encouraged by the potential of porphyrazines, we designed a A_3_B, push–pull porphyrazine (**TT112**, Figure 1) containing six alkyl functions directly attached to three of the pyrrole units and an isoindolecarboxylic moiety as the fourth unit [23]. **TT112** revealed to be a suitable organic photosensitizer for DSSCs, achieving a PCE of 3.42% under full sun illumination (AM1.5G) when incorporated in this type of device. Indeed, the observed efficiency was comparable to that found for the corresponding Pc **TT1** (Figure 1) under the same experimental conditions (η = 3.65%) [23,24,25]. 

Based on this preliminary result, we next prepared a series of A_3_B, panchromatic, push–pull porphyrazines **1a**–**e** (Figure 1) maintaining the electron-withdrawing B moiety of **TT112**, while the A subunits consisted of pyrrole rings substituted with a variety of electron-donor functions [26], and we verified their strong interaction with the aromatic core, as well as their influence in the optical and redox properties of the dyes [27]. Sensitizers **1a**–**e** led to reasonably good fill factor values. However, the power conversion efficiencies were lower than 1%, presumably due to a poor electron injection from the LUMO of the dyes towards the conduction band of the semiconductor [27].

We have also used **TT112** as a light harvester to fill the absorption gap of a pentacene dimer (Pnc_2_), by preparing a Pz–Pnc_2_ conjugate made up of the two chromophores for its application in singlet fission. The panchromatic array underwent quantitative intramolecular Förster resonance energy transfer (i-FRET), from the Pz singlet excited state to the Pnc_2_ ground state, populating the Pnc_2_ singlet excited state, which was finally converted into a pair of correlated triplet excited states [28]. 

Here, we report the synthesis and characterization of a new series of Pzs of A_3_B type, which structure preserves the A unit of **TT112**, while the B unit consists of a pyrrole ring connected to a carboxylic acid function either directly, or through linkers of a different nature. The use of different motifs as the B unit allows modulation of the donor–acceptor character of the dye, as well as the distance and the orientation of the acid function with respect to the macrocycle, these characteristics being central in the performance of the dye [29,30]. Push–pull, donor-π-acceptor dyes, containing carboxylic and other polar functions, find applications as photosensitizers in different fields [31,32,33,34,35,36,37].

## 2. Results

### 2.1. Synthesis

**Scheme 1:**
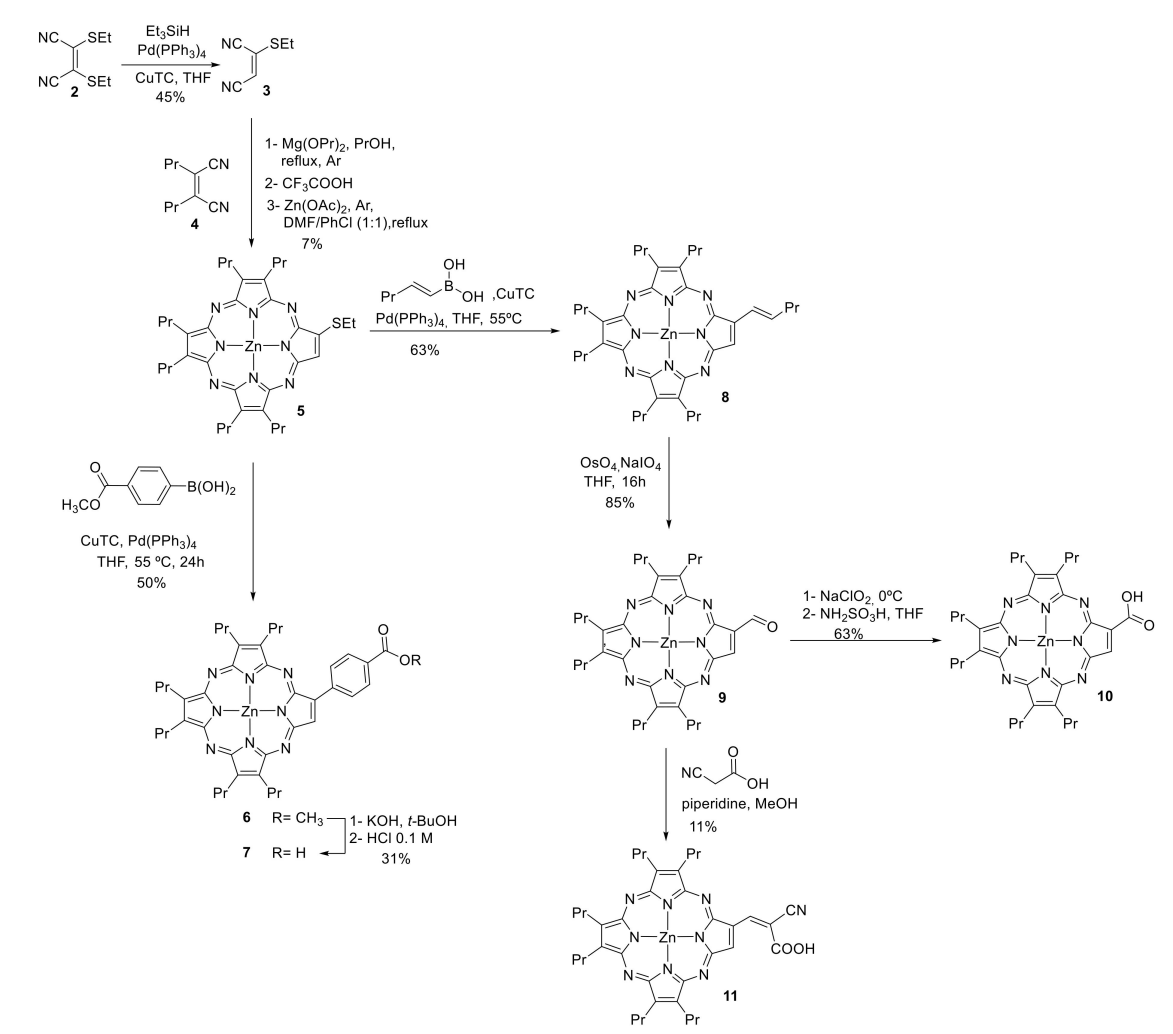
Synthesis of push–pull Pzs **7**, **10**, and **11**.

The syntheses of the Pzs **7**, **10**, and **11**, bearing a carboxylic acid function at the B subunit, are represented in Scheme 1. The key steps consist in Liebeskind–Srogl coupling reactions [38] of a common A_3_B Pz precursor **5**, bearing a thioether group, with appropriate boronic acid derivatives. For this purpose, we prepared the monothioethylmaleonitrile **3** by controlled desulfuration reaction of one of the thioethyl groups in maleonitrile **2**, using the so-called Fukuyama reaction conditions (Scheme 1) [39,40,41,42,43]. Treatment of bis-thioethylmaleonitrile with 1.5 equivalents of CuTC and triethylsilane afforded a mixture of the starting material, the monothioethyl and the unsubstituted maleonitrile. After separation by column chromatography, monothioethylmaleonitrile **3** was obtained as a yellow oil in a fair 45 % yield.

Monothioethylporphyrazine **5** was prepared by crossover Lindstead macrocyclization of a 3:1 mixture of maleonitriles **3** and **4**, in the presence of freshly prepared magnesium propoxide. This led to a mixture of MgPzs, which was subsequently demetallated with TFA. 

Further reaction of the free-base mixture of Pzs with Zn(OAc)_2_ in a refluxing 1:1 mixture of DMF/PhCl, followed by column chromatography on silica gel, yielded the desired A_3_B Pz **5** (Scheme 1) in 7% yield.

**Figure 2:**
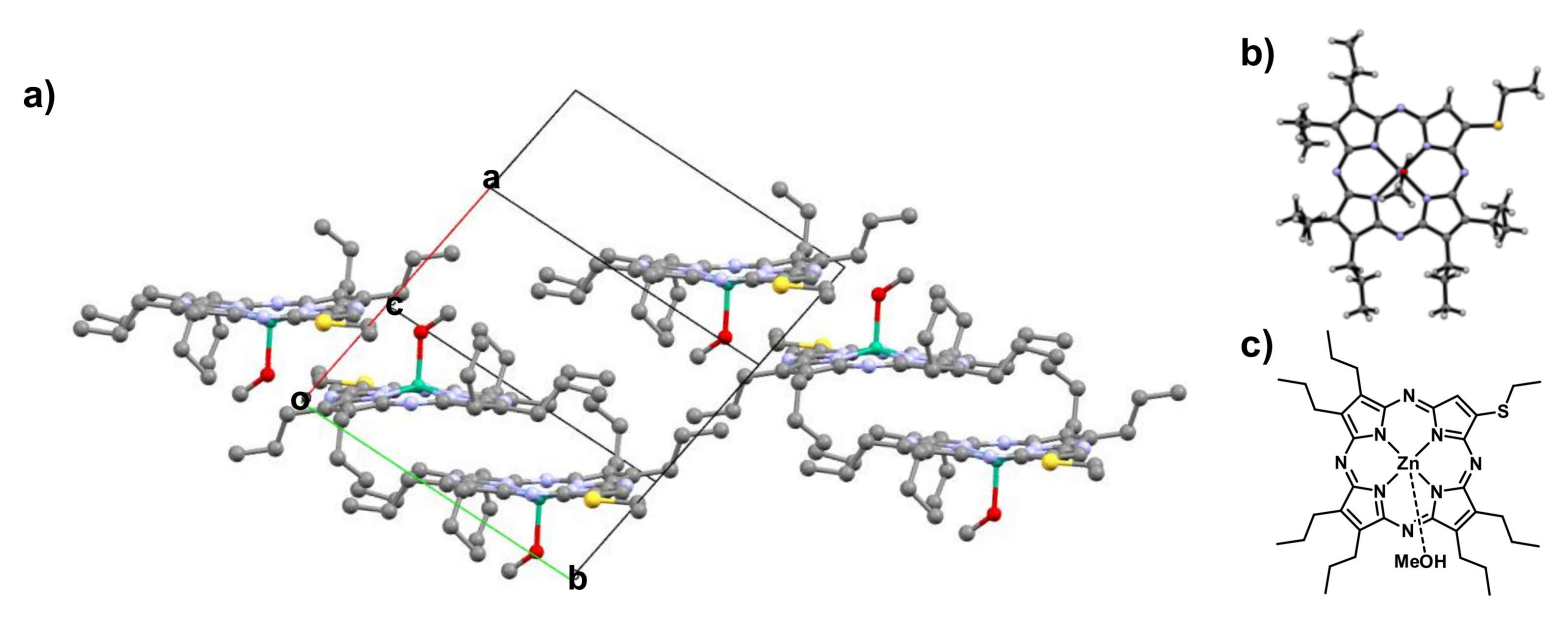
(**a**) Crystal structure of ZnPz **5**, including the unity cell. (**b**) Top view of ZnPz **5**. (**c**) Schematic representation of ZnPz **5**.

A monocrystal of ZnPz **5** was obtained by diffusion of MeOH in a DCM solution of the porphyrazine overnight. The structure was determined by X-Ray diffraction. Figure 2 shows the Zn atom situated above the macrocycle and coordinating a MeOH molecule. The porphyrazine rings are arranged into head-to-head *J*-aggregates, the head being the macrocyclic face to which the axial ligand is attached. The porphyrazines present π–π interactions with the molecules directly above and below in the same staggered column, and Van der Waals interactions between the different columns. The refined crystallographic parameters are given in Table S1. 

Pz **7** contains a *p-*phenylene spacer between the Pz macrocycle and the carboxylic acid function. For its preparation, ZnPz **5** was treated with 4-methoxycarbonylphenylboronic acid in the presence of CuTC, affording **6** (Scheme 1). Hydrolysis of **6** was accomplished by reaction with KOH in *t*-BuOH, followed by acidification with a diluted solution of HCl (Scheme 1), leading to the acid **7** with an overall yield of 15% from **5**. Figure S1 shows the ^1^H NMR spectra of Pzs **6** and **7**. The methoxy group of **6** appears as a singlet at 3.99 ppm, which disappears in the spectrum of **7**. Two doublets at 9.48 and 8.41 ppm (*J* = 8.1 Hz) account for the benzene protons, and the proton corresponding to the *β*-position of the pyrrole ring appears at 9.60 ppm. The MS spectra of **6** and **7** show clusters at *m/z* = 762–769 and *m/z* = 748–755 assigned to the molecular ions of **6** and **7**, respectively, while the corresponding ions appear at *m/z* = 762.3343 and 748.3186, respectively, in HRMS. 

Pz **10** displays the simplest chemical structure, preserving the substitution of **TT112** at the A units, while the B unit contains a carboxylic acid directly attached to the *β*-position of the pyrrole ring. The synthesis of Pz **10** was accomplished by oxidative cleavage of a pentenyl substituted intermediate **8**, followed by oxidation. The preparation of **8** was first essayed from the commercially available 1-penten-1-ylboronic acid, by applying the reaction conditions previously described in our group, that is, using three equivalents of arylboronic acid and CuTC, and 10% mol of palladium catalyst per thioether unit [26]. Under these conditions, we also detected by mass spectroscopy the transmetallated CuPz accompanying ZnPz **8**. Attempts to remove the copper atom from the central cavity with TFA were unsuccessful. In order to minimize the transmetallation reaction, we reduced the amount of CuTC added to 1.2 equivalents. Under these conditions, we were able to isolate **8** in good yields. Oxidation of the vinyl double bond of ZnPz **8** was performed by reaction of the macrocycle with OsO_4_ and NaIO_4_ (Scheme 1) [44]. ZnPz **9** was thus obtained in 85% yield. Further oxidation of ZnPz **9** with NaClO_2_ and sulfamic acid produced the carboxylic acid **10** in 63% yield. The ^1^H-NMR spectra of Pzs **5**, **8**, **9** and **10** (Figure S2) display similar aliphatic signals corresponding to the peripheral propyl groups. **5** also shows in the aliphatic region a quartet at 3.73 ppm and a triplet at 1.71 ppm, corresponding to the thioethyl group, together with the H^β^, which appears as a singlet at δ = 8.75 ppm. For **8**, the signals corresponding to the aliphatic part of the pentenyl chain overlap with those of the peripheral propyl groups, while the signals assigned to the vinyl protons appear at δ = 8.00 ppm, very much influenced by the anisotropic Pz current. Furthermore, a signal at δ = 9.00 ppm accounts for the pyrrolic H^β^. Ongoing from **8** to **9**, the electron-withdrawing aldehyde produces a downfield shift on the H^β^, and the effect is even more pronounced in the case of **10**, owing to the presence of the carboxylic acid (δ = 9.50 ppm and δ = 9.65 ppm, respectively). Finally, the signals corresponding to the protons of the aldehyde and the acid are found at around 12.00 and 15.00 ppm, respectively.

ZnPz **11** bears a cyanoacetic group, which has proven to be suitable for the design of Pz- [45] and Pc- [46] based photosensitizers. By introducing this electron-withdrawing moiety we expected to produce an intramolecular dipole that would propitiate orbital partitioning (*OP*). The latter occurs when the HOMO coefficients are high in the donor part of the molecule, while the LUMO orbital is localized in the accepting part of the structure. Orbital partitioning favors charge separation and, hence, electron injection from the LUMO orbital of the photosensitizer to the semiconductor in DSSCs. ZnPz **11**, was prepared from **9** in 11% yield by Knoevenagel condensation with cyanoacetic acid in the presence of piperidine, in refluxing methanol (Scheme 1). The MS spectrum of **11** exhibits a cluster at *m/z* = 723–731 corresponding to the molecular ion [M]^+^. The ^1^H NMR spectrum (Figure S3) shows three singlets at 10.88, 10.19, 10.05 and a broad signal at 9.8 ppm. Upon addition of D_2_O, the signal at 10.88 ppm corresponding to the acid disappears, and the three other singlets emerge. A singlet at 9.54 ppm is assigned to the pyrrolic *β*-proton, and the two other singlets at 10.13 and 10.00 ppm correspond to the olefinic proton in *E* and *Z* configurations. 

**Scheme 2:**
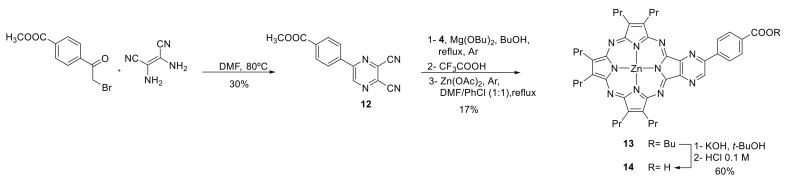
Synthesis of the pyrazinoporphyrazine **14**.

Pz **14** (Scheme 2) has an intrinsic donor–acceptor structure, designed to enhance the dipole character of the Pz macrocycle, through the incorporation of a pyrazine ring annulated to the pyrrole B subunit. The pyrrole[3,4-b]pyrazine moiety was endowed with a carboxylic acid, attached through a phenylene linker. The corresponding pyrazine-dicarbonitrile precursor **12** was prepared in 30% yield by reaction of methyl 4-(2-bromoacetyl)benzoate with diaminomaleonitrile in DMF at 80 °C (Scheme 2). The ^1^H NMR spectrum of **12** (Figure S4) displays the proton corresponding to the pyrazine ring as a singlet at 9.35 ppm, in addition to the two doublets assigned to the *para*-substituted benzene ring between 8.3 and 8.1 ppm. The pyrazinoporphyrazine structure of **14** was assembled by crossover Linstead cyclotetramerization of dinitriles **4** and **12** in a 5:1 ratio, in the presence of Mg(BuO)_2_. Under these conditions, the ester function underwent a transesterification reaction to give the corresponding A_3_B MgPz as a butyl ester derivative, in addition to the A_4_ octapropyl-MgPz as the main products. The mixture was demetallated with TFA and remetallated by treatment with Zn(OAc)_2_, affording the ZnPz **13** in 17%, after column chromatography on silica gel (Scheme 2). The ^1^H NMR spectrum of **13** (Figure S5) shows a singlet at δ = 9.99 ppm corresponding to the pyrazine ring, in addition to the two doublets assigned to the *para*-substituted benzene ring at 8.86 and 8.38 ppm and the butoxy signals in the aliphatic region.

The ZnPz **13** was hydrolyzed by treatment with a solution of KOH in *t*-BuOH, followed by acidification with a diluted solution of HCl, leading to the carboxylic acid **14** in 60% yield, after purification by column chromatography (Scheme 2). The ^1^H NMR spectrum of **14** (Figure S5) evidences the loss of the butyl fragment. MS spectroscopy shows the cluster corresponding to the molecular ion [M]^+^ at *m/z* = 800–806, and HRMS gives a value of 800.3269 for this peak (Figure S12).

### 2.2. Optical Properties

**Figure 3:**
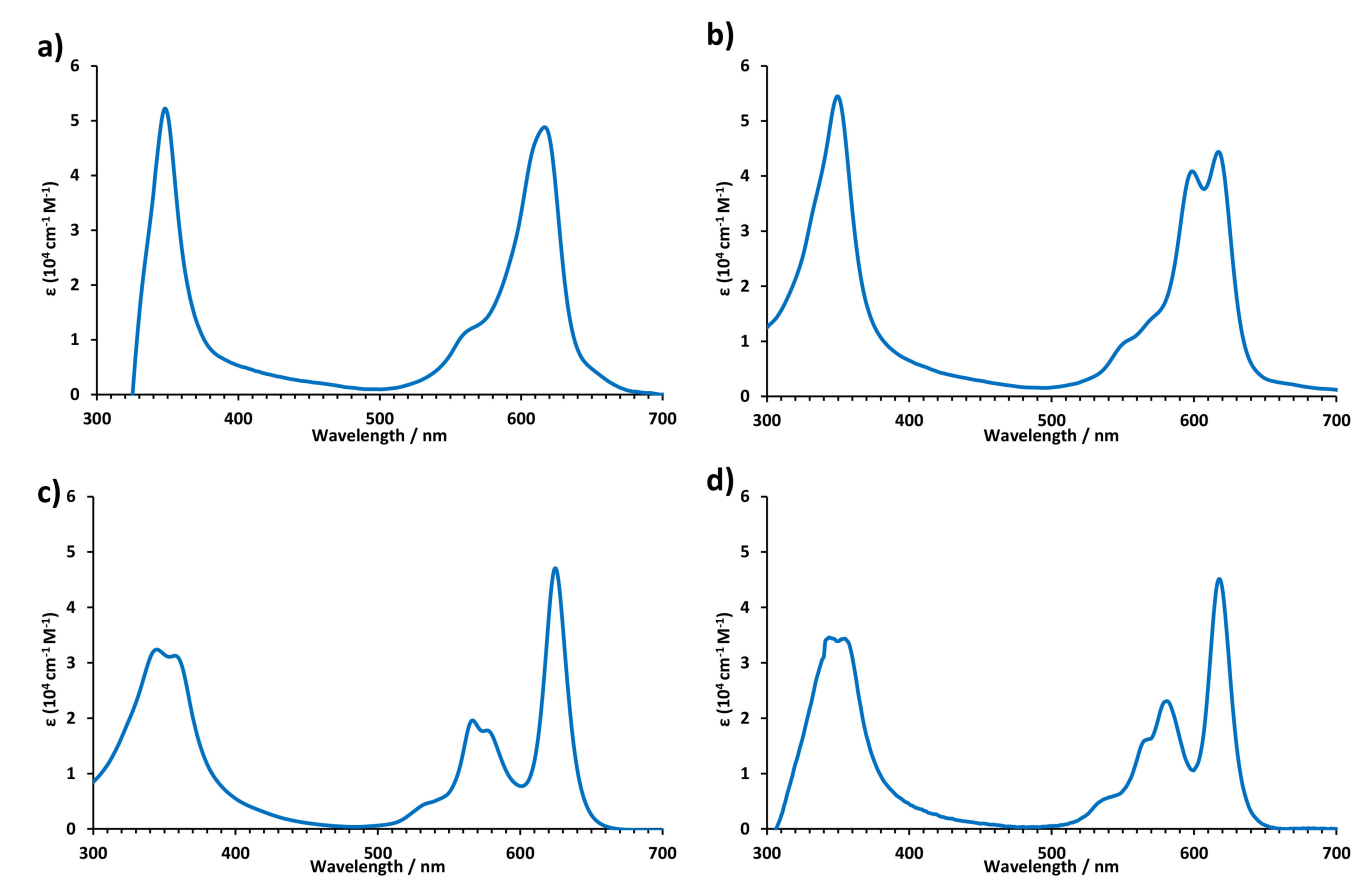
UV-Visible absorption spectra of Pzs (**a**) **5**, (**b**) **8**, (**c**) **9** and (**d**) **10** in THF.

All the prepared ZnPzs display in UV/Vis spectroscopy the typical Soret and Q-bands between 300 and 450 nm and 520 and 650 nm, respectively. The Pz series **5, 8**–**10** exhibit lower molar absorption coefficients in their absorption spectra than **TT112**, probably owing to the decrease in the π-conjugation produced by the removal of the fused benzene ring (Figure 3 and Figure S13). The conversion of the thioether in Pz **5** into the acid in Pz **10** produces a reduction in the symmetry of the Pz, which is reflected by a splitting of the *Q*-band that is already manifest for pentenyl-Pz **8** (~20 nm), maximizes for Pz **9** (~55 nm) and decreases again for Pz **10** (~35 nm). The lowest energy absorption appears in all cases in a range between 615 and 625 nm. The *B*-band also splits in the spectra of Pzs **9** and **10**, and is centered at ~350 nm for all Pzs.

**Figure 4:**
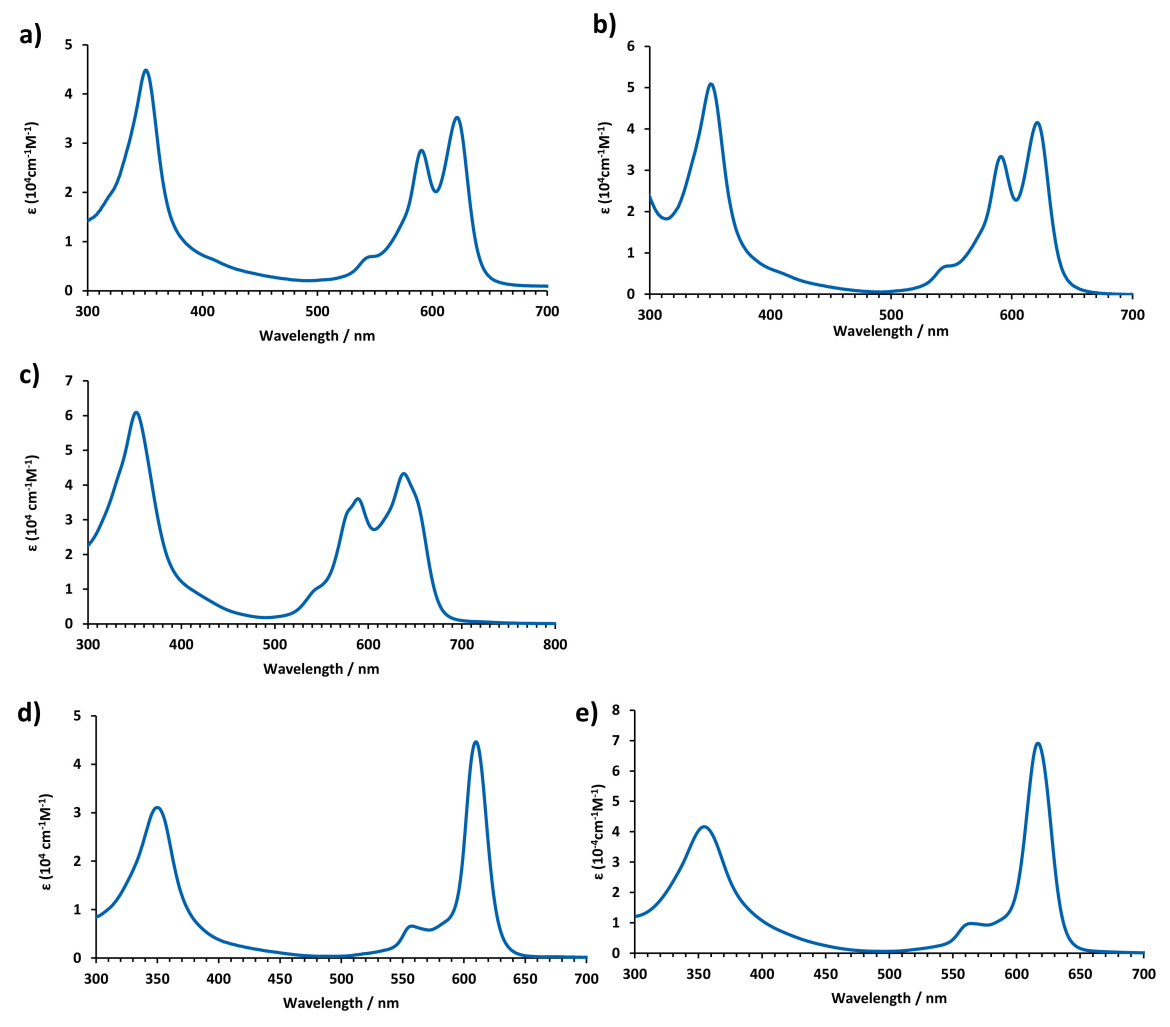
UV-Visible absorption spectra of Pzs (**a**) **6**, (**b**) **7**, (**c**) **11**, (**d**) **13** and (**e**) **14** in THF.

Except pyrazino-Pzs **13** and **14**, which display a single Q-band, the rest of the series also show splitting of their Q-bands with Q_x_ and Q_y_ values at 591 and 622 nm for **7**, and 654(638) and 589(578) nm for the Pzs containing the cyanoacetic acid **11**, respectively (Figure 4 and Figure S14). For **11**, we tentatively assigned λ_Q_ = 654, 589 nm and λ_Q_ = 638, 578 nm to the *E* and Z isomers, respectively, based on the less constrained configuration for the former, which should allow a better conjugation, hence lower-energy Q-band. The single Q-bands of **TT112**, **13** and **14** suggest a weaker interaction of the carboxylic acid functions and their linkers with the macrocyclic core with respect to the other Pzs, where these appendages are directly connected to the pyrrole *β*-position. Very small differences are seen between the ester intermediates and the final carboxylic acids. Thus, both ZnPz **7** and its precursor **6** show absorption maxima at λ_max_ = 622 nm (Figure 4 and Figure S14), while the pyrazinoporphyrazine **14** displays a slight bathochromic shift of the Q-band with respect to its precursor **13** (λ_max_ = 616 and 610 nm, respectively).

Overall, the gradual bathochromic shift of the Q-bands in **10→7→14→11**, seems to arise from the gradual extension of the conjugation ongoing from Pz **10** to **11**. Table 1 summarizes the absorption features of the Pzs. All the Pzs are fluorescent with small Stokes shifts ranging from 25 to 65 meV.

### 2.3. Theoretical Calculations

**Figure 5:**
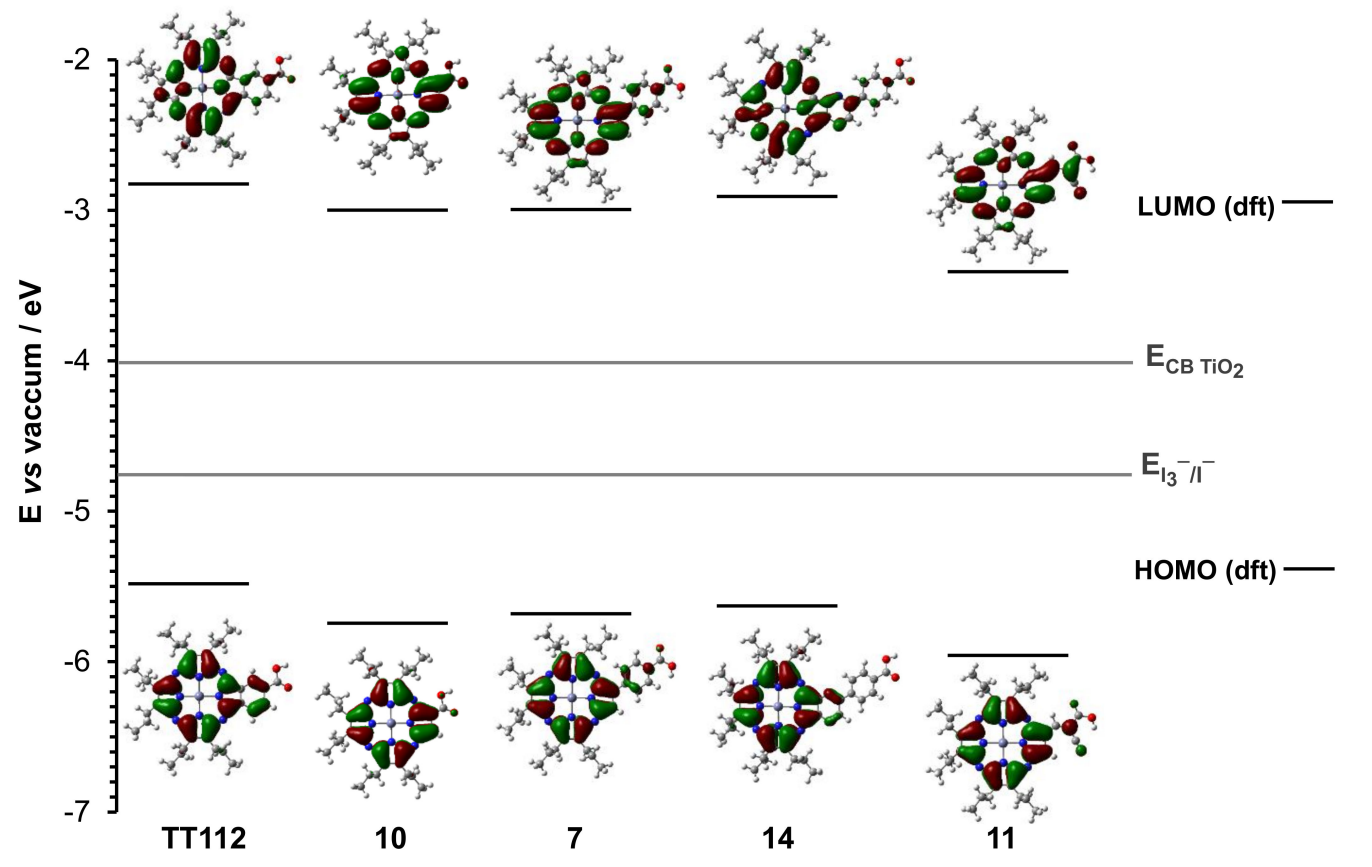
DFT predicted HOMO and LUMO orbitals and frontier orbital energy levels (M06/6-31G (d) for Pzs **TT112, 7, 10**, **11** and **14**. The redox potential of the tri-iodide/iodide couple and the conduction band position of anatase titanium dioxide is represented for information.

In order to provide some insights into the electronic properties of the dyes, DFT calculations were performed using the GAUSSIAN 16, Revision C.01 package [47]. The structures of **TT112**, **7, 10, 11** and **14** were freely optimized in vacuo using the 6-31G(d) basis set, which is frequently used for ZnPcs [48,49]. The M06 functional was chosen owing to it having shown better performance in reproducing X-ray M−Zn bond lengths of metal–porphyrinoids with respect to the widely used B3LYP functional [50]. The same level of theory was used to compute the energies and electronic distributions of the frontier orbitals. For excited states, TD-B3LYP/6-31G(d,p) calculations were performed including the solvent effects (THF) by means of the CPCM conductor-like solvation model. The frontier orbitals predicted for the Pz series are represented in Figure 5.

In general, all Pzs have their HOMO orbital delocalized on the macrocycle, with the delocalization extended over the fused benzene and pyrazine rings in Pzs **TT112** and **14**, respectively. The LUMO orbitals are also delocalized on the macrocycle, in addition to the anchoring subunit, including the carboxylic acid. Hence, no orbital partitioning is observed for any of the derivatives. It is worth noting that delocalization on the carboxylic acid functions is predicted for all the Pzs, but the benzene linkers of **7** and **14** exhibit nodes, which could inhibit the electron injection to an anchored semiconductor such as TiO_2_ nanoparticles.

Table 2 shows the energetic levels for all the calculated frontier orbitals. Relating to DSSCs, the energy levels of tri-iodide/iodide-based redox mediator in electrolyte and the titanium dioxide conduction band are shown. The HOMO energies of **7, 10**, **11** and **14** range between −5.62 and −5.96 eV, similar to those estimated for **TT112** (−5.48 eV), but slightly lower.

Considering these energy data, the regeneration of the I_3_^–^/I^–^ redox pair seems to be feasible in all cases, without significant differences between the dyes. The LUMO energy levels are also very similar, ranging between −2.91 and −3.41 eV, and all of them look satisfactory for an efficient electron injection into the semiconductor. In line with that observed in UV-Visible absorption spectroscopy, the cyanovinyl spacer of **11** produces the largest impact in the Pz core.

The TD-DFT calculation results are in good agreement with the observed relative energy and splitting features of the Q-band absorptions for Pzs **TT112**, **7**, **10**, **11** and **14** (Table S3). As predicted before for other A_3_B porphyrazines [51], theoretical Q bands comprise two transitions from the HOMO to the LUMO and from the HOMO to the LUMO+1 orbitals, with oscillator strengths (*f*) ranging from 0.2 to 0.6. The additional weaker features observed at higher energies are presumably vibronic in character [51]. The calculated peak splitting correlates with the energy difference between the Q_x_ and Q_y_ bands in the UV-Vis spectra. Thus, for Pzs **TT112** and **14** with a single Q-band, the two transitions are predicted closer in energy, at 543, 553 nm and 540, 551 nm, respectively. For porphyrazine **11** the largest splitting is predicted by the two corresponding transitions at 511 and 586 nm. In the middle are Pzs **7** and **10**, with predicted Q-transitions at 523, 554 nm and 509, 539 nm, respectively (Table S3).

Studies on the photovoltaic performances of the Pz dyes were carried out in TiO_2_-based DSSC under different electrolyte compositions. For comparison purpose, benchmark devices made with **TT1** have also been included. The dipping solutions consisted of ca. 0.1 mM of sensitizer in EtOH/THF (7:3) mixture or EtOH for **TT1**, with or without chenodeoxycholic acid (CDCA), using two types of electrolyte. Table 3 and Table S2 summarize the obtained values under standard conditions (A.M. 1.5G).

**Figure 6:**
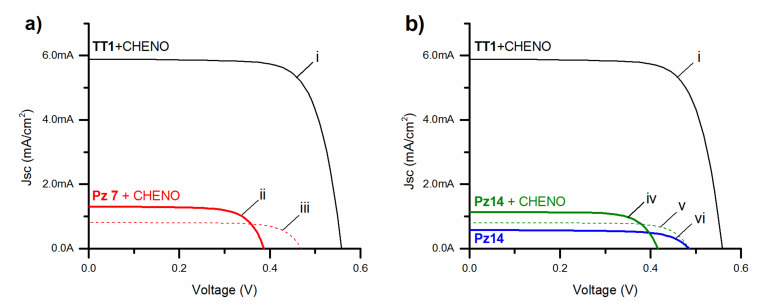
I/V curves of (**a**) Pz **7** and (**b**) Pz **14** using two different electrolytes (A or B), together with the reference cell **TT1**: (i) **TT1**+CHENO/A; (ii) Pz **7**+CHENO/B; (iii) Pz **7**+CHENO/A; (iv) Pz **14**+CHENO/B; (v) Pz **14**+CHENO/A; (vi) Pz **14**/A.

All sensitizers showed poor power conversion efficiencies (<0.5%, see Table 3 and Table S2), falling far below the benchmark (2.76 % obtained with **TT1**). Pzs **10** and **11** showed the lowest efficiencies (<0.1%), mainly due to the small photocurrent generated, with J_SC_ values lower than 0.2 mA∙cm^−2^. The latter could be rationalized in terms of a bad adsorption of these dyes, owing to the proximity of the carboxylic acid to the bulky macrocycle. Pzs **7** and **14**, with a carboxyphenyl anchoring unit, are well chemisorbed onto the TiO_2_ surface, leading to significantly higher J_SC_ values, in the 1 mA∙cm^−2^ range. Hence, they exhibit higher, although still poor PCEs of 0.41% and 0.39%, respectively. (J–V) curves for **7** and **14** are represented in Figure 6.

## 3. Materials and Methods

### 3.1. Instrumentation and Materials

Commercially available solvents and reagents were used without further purification unless otherwise mentioned. Some of the solvents were dried by distillation with Na/benzophenone (THF), or with previously activated molecular sieves (3 or 4 Å), or with an Innovative Technology Inc. MD-4-PS system. Anaerobic reactions were carried out using conventional Schlenk techniques.

Column chromatography was carried out on silica gel Merck-60 (230–400 mesh, 60 Å), and TLC on aluminum sheets precoated with silica gel 60 F254 (E. Merck).

UV-visible absorption spectra were recorded with a JASCO V-660 and Lambda 2 UV–Vis. MS experiments were performed by the Servicio Interdepartamental de Investigación (SIdI) at the Autónoma University of Madrid. EI spectra were obtained from a Waters GCT Agilent Technologies 6890N, FAB spectra were obtained with a Waters VG AutoSpec and MALDI-TOF-MS spectra (Palmer, MA, USA) were obtained from a BRUKER ULTRAFLEX III (Billerica, MA, USA) instrument equipped with a nitrogen laser operating at 337 nm. NMR spectra were recorded with a BRUKER AC-300 (300 MHz) ( Billerica, MA, USA) or a Bruker XRD-500 (500 MHz)( Billerica, MA, USA) instruments. The temperature was actively controlled at 298 K. Chemical shifts were measured in ppm relative to the correspondent deuterated solvent. Carbon chemical shifts were measured downfield from TMS using the resonance of the deuterated solvent as the internal standard. Infrared spectra (IR) were recorded on a Bruker Vector 22 (Billerica, MA, USA).

### 3.2. Synthesis

#### 3.2.1. Thioethylmaleonitrile (**3**)

An oven-dried, degassed Schlenk tube was charged with dithioethylmaleonitrile [52] (300 mg, 1.5 mmol), triethylsilane (0.24 mL, 1.5 mmol), and 8 mL of dry THF under Ar. After a few minutes, copper thiophenecarboxylate (CuTC) [26] (289 mg, 1.5 mmol) and Pd(PPh_3_)_4_ (45mg, 2.5 mol%) were added under Ar. The reaction mixture was heated at 55 °C and monitored by TLC (Heptane/EtOAc 2:1) while small portions of triethylsilane, and Pd(PPh_3_)_4_ were added, until formation of unsubstituted maleonitrile was observed. The mixture was allowed to cool to rt and after, filtered over a Celite pad. The filtrate was washed three times with saturated NaHCO_3_ and twice with H_2_O. The solvent was removed under reduced pressure and the residue purified by column chromatography on silica gel (eluent: Heptane/EtOAc 2:1) to yield a yellowish oil (94 mg, 45%). ^1^H-NMR (300 MHz, CDCl_3_): δ (ppm) = 5.80 (s, 1H), 3.02 (q, *J* = 7.4 Hz, 2H), 1.37 (t, *J* = 7.4 Hz, 3H). ^13^C-NMR (75.5 MHz, CDCl_3_): δ (ppm) = 134.06, 114.31, 111.87, 104.79, 28.23, 13.71. MS (GC-EI): *m/z* = 138.0 [M]^+^. HRMS: *m/z* Calcd for [C_6_H_6_N_2_S]: 138.0252; Found: 138.0248.

#### 3.2.2. [7,8,12,13,17,18-Hexapropyl-2-thioethylporphyrazinato]Zn(II) (**5**)

An oven-dried, 250 mL flask was charged with 100 mL of dry propanol, Mg turnings (120 mg, 4.94 mmol) and a catalytic amount of I_2_ under Ar. After flushing Ar through the system for 5 min, the mixture was heated at 140 °C for 4–6 h until the Mg was completely dissolved and a whitish suspension was observed. After this, dipropylmaleonitrile **4** [53] (704 mg, 5.79 mmol) and monothioethylmaleonitrile **3** (200 mg, 1.45 mmol) were added and the mixture was stirred at 140 °C for 14 h. After allowing the mixture to cool down to rt, the solvent was removed under reduced pressure. An amount of 15 mL of TFA was added to the residue and the solution was stirred at rt in the dark for 1 h. The solution was then carefully poured into a 1:1 ice-water/NH_3_ (30% ac.) mixture, and the resulting precipitate was filtered and washed with water and a 1:1 mixture of water/MeOH. The precipitate was dissolved in 40 mL of a 1:1 mixture of DMF/PhCl and Zn(OAc)_2_ (650 mg, 3.6 mmol) was added. The resulting solution was heated at 140 °C until no remaining starting material was observed. Afterwards, the solvent was removed under reduced pressure. The residue was purified by column chromatography on silica gel (Toluene with 1% THF) to yield a blue solid (65 mg, 7%). ^1^H-NMR (300 MHz, THF-*d_8_*): δ (ppm) = 8.75 (s, 1H), 3.99–3.85 (m, 12H), 3.72 (q, *J* = 7.4 Hz, 2H), 2.38 (m, 12H), 1.79 (t, *J* = 7.4 Hz, 3H), 1.28 (m, 18H). ^13^C-NMR (75.5 MHz, THF-*d_8_*): δ (ppm) = 159.46, 159.37, 159.05, 158.94, 158.38, 155.70, 149.09, 145.21, 145.03, 144.84, 144.71, 124.91, 67.57, 30.80, 29.16, 29.03, 27.89, 26.62, 26.55, 15.22, 14.75. FT-IR (KBr): *ν* (cm^−1^) = 2958.3, 2930.3, 2870.5, 1724.0, 1633.4, 1490.7, 1464.7, 1371.1, 1315.2, 1264.1, 1235.2, 1154.1, 1117.5, 1085.7, 1071.6, 1036.6, 1015.3, 950.7, 890.0, 861.1, 794.5, 770.4, 757.9, 730.7, 573.7. UV-vis (THF): λ_max_ (nm) (log ε (dm^3^ mol^−1^ cm^−1^)) = 619 (4.69), 567(sh), 350 (4.72). MS (MALDI-TOF, DCTB): *m/z* = 688.4 [M]^+^. HRMS: *m/z* Calcd for [C_36_H_48_N_8_SZn]: 688.3009; Found: 688.3001.

#### 3.2.3. [7,8,12,13,17,18-Hexapropyl-2-(4-methoxycarbonylphenyl)porphyrazinato]Zn(II) (**6**)

Pz **5** (91 mg, 0,13 mmol), 4-methoxycarbonylphenylboronic acid (71 mg, 0.4 mmol), Pd(PPh_3_)_4_ (15 mg, 0.013 mmol), CuTC (30 mg, 0.16 mmol) and THF (4.5 mL) were placed under argon in a Schlenk tube, and the mixture was stirred at 60 °C for 24h. After cooling at rt, the solvent was evaporated and the crude was chromatographed on silica gel (Toluene/THF 98:2) affording **6** as a blue solid (53 mg, 53%). ^1^H RMN (300 MHz, THF-*d*_8_): δ(ppm) = 9.60 (s, 1H), 9.48 (d, *J* = 8.2 Hz, 2H), 8.41 (d, *J* = 8.1 Hz, 2H), 3.99 (s, 3H), 3.98–3.88 (m, 12H), 2.44–2.35 (m, 12H), 1.30 (t, *J* = 7.3 Hz, 18H). FT-IR *ν* (cm^−1^): 2953, 2874, 1718, 1459, 1312, 1369, 1231, 1151, 1010, 948, 756. UV-Vis (THF), λ_max_ (nm) (log ε): 622 (4.5), 591 (4.4), 544 (3.8), 415, (sh), 351 (4.6). Fluorescence (THF), λ_em_ (nm) = 643. MS (MALDI-TOF, DCTB): *m/z* = 762.3–769.3 [M]^+^. HRMS: *m/z* Calcd for [C_42_H_50_N_8_O_2_Zn]: 762.3343; found: 762.3343.

#### 3.2.4. [7,8,12,13,17,18-Hexapropyl-2-(4-carboxyphenyl)porphyrazinato]Zn(II) (**7**)

To a solution of Pz **6** (47 mg, 0.06 mmol) in *t*-BuOH (62 mL) KOH 85% (69 mg 1.2 mmol) was added and the mixture was stirred at 40 °C and monitored by TLC until the conversion was complete. HCl 0.1 M (62 mL) was added and the solution was extracted with DCM (3 × 50 mL). The organic extracts were collected and washed with water (2 × 30 mL). The solvent was rotary evaporated, and the residue was chromatographed on silica gel (Tol/THF 1:1 and then THF-MeOH 1:1), affording **7** as a blue solid (28 mg, 60%). ^1^H RMN (300 MHz, THF-*d*_8_): δ (ppm) = 9.59 (s, 1H), 9.46 (d, *J* = 8.0 Hz, 2H), 8.43 (d, *J* = 8.0 Hz, 2H), 3.94 (m, 12H), 2.40 (m, 12H), 1.30 (m, 18H). FT-IR: *ν* (cm^−1^): 3311-2527, 1718, 1667, 1604, 1425, 1274, 946, 752. UV-Vis (THF), λ_max_ (nm) (log ε): 622 (4.6), 591 (4.5), 544 (3.8), 414 (sh), 351 (4.7). Fluorescence (THF), λ_em_ (nm)= 637. MS (MALDI-TOF, DCTB): *m/z* = 748.3–755.3 [M]^+^. HRMS: *m/z* Calcd. for [C_41_H_48_N_8_O_2_Zn]: 748.3192; found: 748.3186.

#### 3.2.5. [7,8,12,13,17,18-Hexapropyl-2-pent-1-enylporphyrazinato]Zn(II) (**8**)

An oven-dried flask containing Pz **5** (40 mg, 0.058 mmol), 1-pent-1-enylboronic acid (20 mg, 0.176 mmol), [Pd(PPh_3_)_4_] (7 mg, 0.006 mmol;), and CuTC (14.6 mg, 0.076 mmol) were added and the mixture was purged with Ar, and then charged with dry THF (2 mL). The mixture was stirred at 60 °C for 20 h under Ar atmosphere. After cooling to room temperature, the crude was passed through a silica gel pad. The filtrate was evaporated and the residue was purified by chromatography on silica gel (eluent Toluene/THF 99:1). The products were recrystallized from CH_2_Cl_2_ and *n*-hexane. ^1^H-NMR (300 MHz, THF-*d_8_*): δ (ppm) = 9.00 (s, 1H), 7.96 (m, 2H), 4.20–3.77 (m, 12H), 2.47–2.17 (m, 12H), 1.88 (m, 2H), 1.25 (m, 23H). ^13^C-NMR (75.5 MHz, THF-*d_8_*): δ (ppm) = 159.99, 159.88, 159.70, 159.45, 159.15, 158.74, 158.45, 156.73, 145.37, 145.15, 145.03, 144.87, 144.67, 138.67, 127.68, 124.56, 37.55, 33.03, 30.80, 30.47, 29.17, 29.06, 23.94, 23.73, 15.26, 15.21, 14.60. FT-IR (KBr): *ν* (cm^−1^) = 2961.3, 2934.3, 2870.5, 1717.4, 1663.3, 1447.2, 1261.7, 1207.7, 1122.2, 876.7, 673.6, 619.4. UV-vis (THF): λ_max_ (nm) (log ε (dm^3^ mol^−1^ cm^−1^)) = 617 (4.64), 599 (4.60), 570 (sh), 555 (sh), 350 (4.73). MS (MALDI-TOF, DCTB): *m/z* = 696.4 [M]^+^. HRMS: *m/z* Calcd for [C_39_H_52_N_8_Zn]: 696.3601; Found: 696.3609.

#### 3.2.6. [7,8,12,13,17,18-Hexapropyl-2-formylporphyrazinato]Zn(II) (**9**)

To a suspension of OsO_4_ (364 mg, 1% wt of polyvinylpyridine) and Pz **8** (10 mg, 0.014 mmol) in 15 mL of THF a saturated aqueous solution of NaIO_4_ (5 mL) was added dropwise at rt. The reaction mixture was stirred for 16h at that temperature, then filtered over Celite, and the filtrate was evaporated under reduced pressure. The crude product was purified by flash column chromatography (eluent: Toluene/THF 98:2), affording Pz **9** as a blue solid (8 mg, 85%). ^1^H-NMR (300 MHz, THF-*d_8_*): δ (ppm) = 11.84 (s, 1H), 9.52 (s, 1H), 3.87 (m, 12H), 2.38 (m, 12H), 1.30 (m, 18H). ^13^C-NMR (75.5 MHz, THF-*d_8_*): δ (ppm) = 188.42, 163.57, 163.38, 163.22, 162.87, 159.09, 158.94, 154.70, 154.10, 147.13, 146.78, 146.68, 145.39, 145.09, 138.88, 132.16, 29.05, 28.97, 26.46, 26.40, 26.37, 15.21, 15.14. FT-IR (KBr): *ν* (cm^−1^) = 2961.3, 2935.0, 2866.3, 1718.4, 1664.2, 1516.4, 1448.2, 1273.5, 1205.5, 1124.2, 1097.2, 775.7, 673.6, 627.5. UV-vis (THF): λ_max_ (nm) (log ε (dm^3^ mol^−1^ cm^−1^)) = 625 (4.67), 577 (4.25), 567 (sh), 539 (4.29), 358 (4.50), 345 (4.51). MS (MALDI-TOF, DCTB): *m/z* = 656.4 [M]^+^. HRMS: *m/z* Calcd for [C_35_H_44_N_8_OZn]: 656.2924; Found: 656.2918.

#### 3.2.7. [7,8,12,13,17,18-Hexapropyl-2-carboxyporphyrazinato]Zn(II) (**10**)

To a solution of formylPz **9** (8 mg, 0.012 mmol) in THF (2 mL) at 0 °C, a solution of NaClO_2_ (27 mg, 0.024 mmol) in water (0.5 mL, Milli-Q grade) was added dropwise. Then, a solution of sulfamic acid (3 mg, 0.03 mmol) in water (0.6 mL, Milli-Q grade) was added in one portion. After the starting material disappeared (*ca.* 10 min), the solution was poured into aqueous HCl (5 mL, 1 M) and a greenish solid precipitated. The solid was filtered, dissolved in DCM, washed with water and dried with MgSO_4_. The crude product was purified by flash column chromatography (eluent: Toluene/THF 2:1), affording Pz **10** as a blue solid (5 mg, 63%). ^1^H-NMR (300 MHz, THF-*d_8_*): δ (ppm) = 14.75 (br s, 1H), 9.64 (s, 1H), 3.87 (m, 12H), 2.37 (m, 12H), 1.30 (m, 18H). ^13^C-NMR (75.5 MHz, THF-*d_8_*): δ (ppm) = 163.87, 163.45, 162.99, 162.77, 162.59, 159.50, 157.97, 154.42, 152.43, 147.25, 147.01, 146.77, 146.13, 145.53, 136.81, 132.58, 33.03, 30.79, 30.46, 29.07, 15.21, 15.14, 14.58. FT-IR (KBr): *ν* (cm^−1^) = 3383.6 (br), 2968.4, 2928, 2860.5, 1713.3, 1665.3, 1097.2, 885.6, 777.5, 615.2. UV-vis (THF): λ_max_ (nm) (log ε (dm^3^ mol^−1^ cm^−1^) = 618 (4.65), 581 (4.36), 570 (sh), 544 (3.83), 355 (4.54), 344 (4.54). Fluorescence (THF), λ_em_ (nm) = 625. MS (MALDI-TOF, DCTB): *m/z* = 672.4 [M]^+^. HRMS: *m/z* Calcd for [C_35_H_44_N_8_O_2_Zn]: 672.2873; Found: 672.2846.

#### 3.2.8. [7,8,12,13,17,18-Hexapropyl-2-(2-carboxy-2-cyanovinyl)porphyrazinato]Zn(II) (**11**)

A solution of Pz **9** (19 mg, 0.03 mmol), cyanoacetic acid (12 mg, 0.14 mmol, 5.0 eq.) and piperidine (67 µL, 0.68 mmol, 24 eq.) in MeOH (2 mL) was refluxed for 10 h. After cooling at room temperature, the solvent was rotary evaporated, and the residue was dissolved in CH_2_Cl_2_ (100 mL). The solution was extracted with H_2_O (200 mL), shaking the mixture vigorously and fixing the pH of the aqueous phase at 2 with H_3_PO_4_ 2 M (7.0 mL). The organic phase was washed once more with water (200 mL) containing H_3_PO_4_ 2M (1.0 mL, pH = 2). The solvent was rotary evaporated, and the residue was chromatographed on silica gel (Tol/THF 2:1) and then THF/MeOH (1:1) affording **11** as a blue solid (2.3 mg, 11%). ^1^H RMN (300 MHz, THF-*d_8_* + D_2_O): δ (ppm) = 10.13 (s, 1H), 10.00 (s, 1H), 9.54 (s, 1H), 3.87 (m, 12H), 2.36 (m, 12H), 1.28 (m, 18H). UV-vis (THF): λ_max_ (nm) (log ε (dm^3^ mol^−1^ cm^−1^)) = 654 (sh), 638 (4.6), 589 (4.5), 578(sh), 541 (sh), 352 (4.8). MS (MALDI-TOF, DCTB): *m/z* = 723-731 [M]^+^.

#### 3.2.9. Methyl 4-(5,6-dicyanopyrazine-2-yl)benzoate (**12**)

A solution of methyl 4-(2-bromoacetyl) benzoato (400 mg, 1.5 mmol) and diaminomaleonitrile (168 mg, 1.5 mmol) in DMF (4 mL) was stirred at 80 °C for 4h. The solvent was evaporated at reduced pressure and the residue was chromatographed on silica gel (heptane/AcOEt 7:3) to give **12** (123 mg, 30%). ^1^H RMN (300 MHz, CDCl_3_): δ (ppm) = 9.35 (s, 1H), 8.30–8.14 (m, 4H), 3.99 (s, 3H). ^13^C RMN (75.5 MHz, CDCl_3_): δ (ppm) = 165.9, 153.8, 144.3, 136.3, 133.9, 133.3, 131.6, 130.8, 128.0, 112.9, 112.8, 52.6. FT-IR *ν* (cm^−1^): 2953, 2916, 2845, 2238, 1710, 1548, 1435, 1278, 1011, 864, 771, 698. MS (ESI-MeOH + NaI): *m/z* = 287.0539 [M + Na]^+^.

#### 3.2.10. [7,8,12,13,17,18-Hexapropyl-(4-butoxycarbonylphenyl[2,3b]-pyrazine)porphyrazinato] Zn(II) (**13**)

Mg turnings (44 mg, 1.8 mmol) and a cheap of iodine were heated in butanol (17 mL) at reflux under argon for 8h. Dipropylmaleonitrile **4** (491 mg, 3.0 mmol) and **12** (120 mg, 0.6 mmol) were added and the mixture was heated for additional 24 h. After cooling at rt, the solvent was rotary evaporated, and the residue was dissolved in TFA (12 mL) and stirred at rt for 1 h. The mixture was poured in a 1:1 mixture of ice/NH_3_ (30% ac.) and the resulting precipitate was filtered, washed with water and then with a 1:1 mixture of methanol–water. After drying the blue solid, it was placed in a flask and dissolved in a 1:1 mixture of DMF/PhCl (25 mL). Zn(OAc)_2_ (275 mg, 1.5 mmol) was added and the mixture was heated at 140 °C for 24 h. the solvent was rotary evaporated and the residue was chromatographed on silica gel (CHCl_3_/Tol 7:3) slowly increasing the polarity. Compound **13** was obtained as a blue solid (66 mg, 17 %). ^1^H-RMN (300 MHz, THF-d_8_): δ (ppm) = 9.99 (s, 1H), 8.86 (d, *J* = 8.3 Hz, 2H), 8.38 (d, *J* = 8.2 Hz, 2H), 4.43 (t, *J* = 6.6 Hz, 2H), 4.10–3.90 (m, 14H), 2.53–2.33 (m, 12H), 1.86 (dd, *J* = 8.7, 6.3 Hz, 2H), 1.66–1.56 (m, 2H), 1.32 (m, 18H), 1.08 (t, *J* = 7.4 Hz, 3H). FT-IR *ν* (cm^−1^): 2957, 1720, 1464, 1271, 1105, 1015, 948, 752. UV-Vis (THF), λ_max_ (nm) (log ε): 610 (4.6), 582 (sh), 558 (3.8), 350 (4.5). Fluorescence (THF), λ_em_ (nm) = 622. MS (MALDI-TOF, DCTB): *m/z* = 856.4–863.4 [M]^+^. HRMS: *m/z* Calcd. for [C_47_H_56_N_10_O_2_Zn]: 856.3874; found: 856.3869.

#### 3.2.11. [7,8,12,13,17,18-Hexapropyl-(4-carboxyphenyl[2,3b]-pyrazine)porphyrazinato]Zn(II) (**14**)

To a solution of Pz **13** (55 mg, 0.06 mmol), in *t*-BuOH (64 mL), KOH 85% (71.9 mg 1.28 mmol) was added, and the mixture was stirred at 40 °C and monitored by TLC until the reaction was complete. HCl 0.1 M (60 mL) was added, and the mixture was extracted with DCM (3 × 20 mL). The organic extracts were collected and washed with H_2_O (2 × 10 mL). The solvent was rotary evaporated, and the residue was chromatographed on silica gel (Tol/THF 2:1 and then THF/MeOH 1:1), affording **14** as a blue solid (30 mg, 60%). ^1^H RMN (300 MHz, DMSO-d_6_): δ (ppm) = 10.00 (s, 2H), 8.58 (m, 2H), 8.33 (d, *J* = 1.3 Hz, 2H), 3.86 (d, *J* = 8.3 Hz, 12H), 2.37 (s, 12H), 1.33–1.18 (m, 18H). FT-IR *ν* (cm^−1^): 3291–2525, 1685, 1608, 1462, 1284, 1015, 931, 728. UV-Vis (THF), λ_max_ (nm) (log ε): 616 (4.8), 564 (4.0), 354 (4.6). Fluorescence (THF), λ_em_ (nm) = 631. MS (MALDI-TOF, DCTB): *m/z* = 800.3–806.3 [M]^+^. HRMS: *m/z* Calcd. for [C_43_H_48_N_10_O_2_Zn]: 800.3248; found: 800.3269.

### 3.3. Device Preparation

A transparent fluorine-doped tin oxide conducting glass (NSG10 or TEC8) was cleaned using ethanol and water followed by an ultrasonic cleaning in Deconnex^TM^ solution for 30 min. The electrodes were then washed with water and ethanol. To remove the organics, a further thermal treatment was done at 500 °C for 30 min. The clean FTO glass was treated twice with TiCl_4_ (40 mM, 30 min, 75 °C). Two different TiO_2_ pastes (transparent layer and scattering layer, Dyesol) were screen printed on to the TiCl_4_ pre-treated electrode and followed a series sintering step. The transparent layer and scattering layers consisted of 20 nm particles (with a pore diameter of 32 nm) and 400 nm particles, respectively. The photoanodes were further treated with TiCl_4_ following the steps described above. The TiO_2_ electrodes were dried at 500 °C for 30 min prior to the sensitization in the dye solution. The dye solutions consisted of 0.1 mM of the sensitizer (with or without CHENO) in EtOH/THF (7:3) for **Pzs** or in absolute EtOH for **TT1**. After 18h of dipping, the electrodes were washed with EtOH to remove the loosely bound dye molecules. The counter electrodes were made by drop casting of isopropanolic solution of H_2_PtCl_6_ (5 mM) onto a pre-cleaned FTO glass (TEC7) and the electrodes were fired at 410 °C for 30 min. The sensitized photoanode and counter electrode were melt-sealed using a 25 μm polymeric spacer (Surlyn^TM^). The electrolyte in acetonitrile was then injected by the vacuum backfilling process through a hole drilled at the side of the counter electrode. The electrolyte composition was DMII 0.6 M, GuNCS 0.05 M, I_2_ 0.04 M, TBP 0.28 M and LiI (0.1 M for electrolyte A or 0.5 M for electrolyte B) in AcCN. The cell fabrication was completed by melt sealing the hole with a piece of glass and soldering the metal solder to make contacts.

### 3.4. Photovoltaic Characterization

A 450W xenon lamp was used as a light source. The spectral output of the lamp was filtered using a sunlight filter to reduce the mismatch between the simulated and actual solar spectrum. The current-voltage characteristics of the cell were recorded with a Keithley model 2400 digital source meter. The photo-active area of 0.159 cm^2^ was defined by a square black metal mask.

## 4. Conclusions

A series of A_3_B Pzs, containing six *n*-propyl chains directly attached to the macrocyclic core as the donor A units, and a B acceptor subunit endowed with a carboxylic acid function, was prepared. The carboxylic function was attached directly to the β-position of the pyrrole unit (**10**), or through different spacers, namely, cyanovinyl (**11**) and phenylene (**7**) moieties. In addition, a pyrazinoporphyrazine with intrinsic push–pull character, and bearing a carboxyphenyl substituent (**14**) was synthesized. The effects of the different B subunits in the properties of the low-symmetric macrocycles have been studied. Introduction of the cyanovinyl spacer produces the strongest effect in the frontier orbitals, decreasing their energetic levels. This is also evidenced in UV-Visible absorption spectroscopy through a large splitting of the Q-band in **11**, arising from the strong desymmetrization of the macrocycle, induced by the cyanovinyl moiety.

The performance of the four Pzs as photosensitizers in DSSCs has also been investigated, all of them showing relatively low conversion performances. Comparison of the values obtained for **7**, **10**, **11** and **14** with those obtained previously for **TT112** suggests that the electronic nature of the B unit carrying the anchoring carboxylic acid must have a strong influence in both adsorption and electron injection processes. This is in agreement with our previous observations on the high sensitivity of the PCEs to Pz peripheral substitution [27]. What is more, anchoring the dye to the semiconductor might disturb the electronic character of the Pz to a higher extent than in other types of porphyrinoids. Overall, the work brings out once more the powerful effect of peripheral functionalization in the electronic properties of Pzs and points out the narrow room for maneuver in the design of porphyrazines for photovoltaic applications [54].

## Figures and Tables

**Table 1:** Absorption properties of Pzs.

Pz	Absorption λ (nm) (*ε* [10^4^ M^−1^cm^−1^])
**TT112** [23]	615 (5.07), 560 (4.25), 346 (4.78)
**5**	619 (4.69), 567 (sh), 350 (4.72).
**6**	622 (4.5), 591 (4.4), 544 (3.8), 415, (sh), 351 (4.6)
**7**	622 (4.6), 591 (4.5), 544 (3.8), 414 (sh), 351 (4.7)
**8**	617 (4.64), 599 (4.60), 570 (sh), 555 (sh), 350 (4.73)
**9**	625 (4.67), 577 (4.25), 567 (sh), 539 (4.29), 358 (4.50), 345 (4.51)
**10**	618 (4.65), 581 (4.36), 570 (sh), 544 (3.83), 355 (4.54), 344 (4.54)
**11**	654 (sh), 638 (4.6), 589 (4.5), 578 (sh), 541 (sh), 352 (4.8)
**13**	610 (4.6), 582 (sh), 558 (3.8), 350 (4.5).
**14**	616 (4.8), 564 (4.0) 354 (4.6)

**Table 2:** Predicted energy levels (eV) of HOMO and LUMO orbitals of Pzs **TT112**, **7, 10**, **11** and **14** by DFT method.

Pz	HOMO (eV vs. Vac.)	LUMO (eV vs. Vac)
**TT112**	−5.48	−2.81
**10**	−5.74	−3.02
**7**	−5.69	−3.00
**14**	−5.62	−2.91
**11**	−5.96	−3.41

**Table 3:** Photovoltaic data of the best DSSCs sensitized with Pz-**7**, **14** and benchmark **TT1** under simulated AM1.5G illumination (~0.9 sun intensity).

Entry	Dye ^a^ (0.1 mM)	[CHENO] ^b^ (mM)	Elect. ^c^ (LiI Conc.)	J_SC_ (mA∙cm^−2^)	V_OC_ (mV)	F.F. (%)	Pin (mW∙cm^−^^2^)	η (%)
(i)	**TT1**	10	A (0.1 M)	5.89	558	74.6	88.9	2.76
(ii)	**7**	10	B (0.5 M)	1.31	387	71.3	89.1	0.41
(iii)	**7**	10	A (0.1 M)	0.806	470	74.3	88.7	0.32
(iv)	**14**	10	B (0.5 M)	1.13	416	73.6	89.1	0.39
(v)	**14**	10	A (0.1 M)	0.797	485	75.2	88.9	0.33
(vi)	**14**	0	A (0.1 M)	0.579	485	69.8	88.9	0.22

^a^ Dye-uptake solutions consisted of 0.1 mM of the sensitizer (with or without CHENO) in EtOH/THF (7:3) for Pzs 7, 14 or EtOH for TT1 (dipping time 18h). ^b^ CHENO was incorporated in the dye-uptake solution. ^c^ Iodine-based electrolytes A and B are composed of 0.6 M 1,3-dimethyl imidazolium iodine, 0.28 M 4-*tert*-butyl pyridine, 0.04 M I_2_, 0.05 M guanidinium thiocyanate in AcCN, and differ only by the concentration of lithium iodide: 0.1 M (A) and 0.5 M (B).

## Data Availability

Data of the compounds are available from the authors.

## Supplementary Materials

The following are available online, Figure S1: 1H-RMN spectrum of **6** (lower) and **7** (upper) inTHF-d8. *BHT, Figure S2: 1H-NMR spectra of Pzs **5**, **8**, **9** and **10** in THF-d8, Figure S3: 1H-RMN spectrum of **11** in THF-d8 (upper) and in THF-d8 + D2O (lower), Figure S4: 1H-RMN spectrum of **12** in CDCl3. *H2O, Figure S5: 1H-RMN Spectrum of **13** (lower) and **14** (upper) in THF-d8., Figure S6: FT-IR spectrum (KBr) of **7**, Figure S7: MS and HRMS spectra (MALDI-TOF) of 7, Figure S8: FT-IR spectrum (KBr) of **10**, Figure S9: MS and HRMS spectra (MALDI-TOF) of **10**, Figure S10: MS spectra (MALDI-TOF) of **11** and isotopic pattern, Figure S11: FT-IR spectrum (KBr) of **14**, Figure S12: MS and HRMS spectra (MALDI-TOF) of **14**, Figure S13: UV-Visible absorption spectra of Pzs **5**, **8**, **9** and **10** in THF, Figure S14: UV-Visible absorption spectra of Pzs **6**, **7**, **11**, **13** and **14** in THF, Table S1: Crystallographic data of Pz 5, Table S2: Pho-tovoltaic data of the DSSC devices made with Pzs and benchmark TT1 adsorbed on 14 mm TiO2 films, Table S3.: Predicted and experimental absorption bands (λmax) in THF, oscillator strengths (f) and dominant electronic transitions, for porphyrazines **TT112**, **7**, **10**, **11** and **14**.
