# Supplementary material for: Tuning the Acceptor Unit of Push–Pull Porphyrazines for Dye-Sensitized Solar Cells
Source: Molecules. 2021 Apr 7;26(8):2129. doi: 10.3390/molecules26082129 (PMC8068076; doi:10.3390/molecules26082129)
Supplement: Supplementary file 1 [file molecules-26-02129-s001.pdf]

Supporting Information

Tuning the Acceptor Unit of Push-Pull Porphyrazines for Dye-Sensitized Solar Cells

Diana-Paola Medina[1], Javier Fernández-Ariza[1], Maxence Urbani[2], Frédéric Sauvage[3], Tomás Torres[1,2,*] and M. Salomé Rodríguez-Morgade[1,*]

[1] Departamento de Química Orgánica, Universidad Autónoma de Madrid, Cantoblanco, 28049 Madrid, Spain
[2] Instituto Madrileño de Estudios Avanzados (IMDEA)-Nanociencia, c/ Faraday, 9, Cantoblanco, 28049 Madrid, Spain
[3] Université de Picardie Jules Verne, Laboratoire de réactivité et chimie des solides, CNRS UMR7314, Hub de l'énergie, 15 rue Baudelocque, 80039 Amiens, France
* Correspondence: tomas.torres@uam.es, salome.rodriguez@uam.es

Figure S1. ^{1}H-RMN spectrum of **6** (lower) and **7** (upper) inTHF-$d_{8.}$ *BHT.

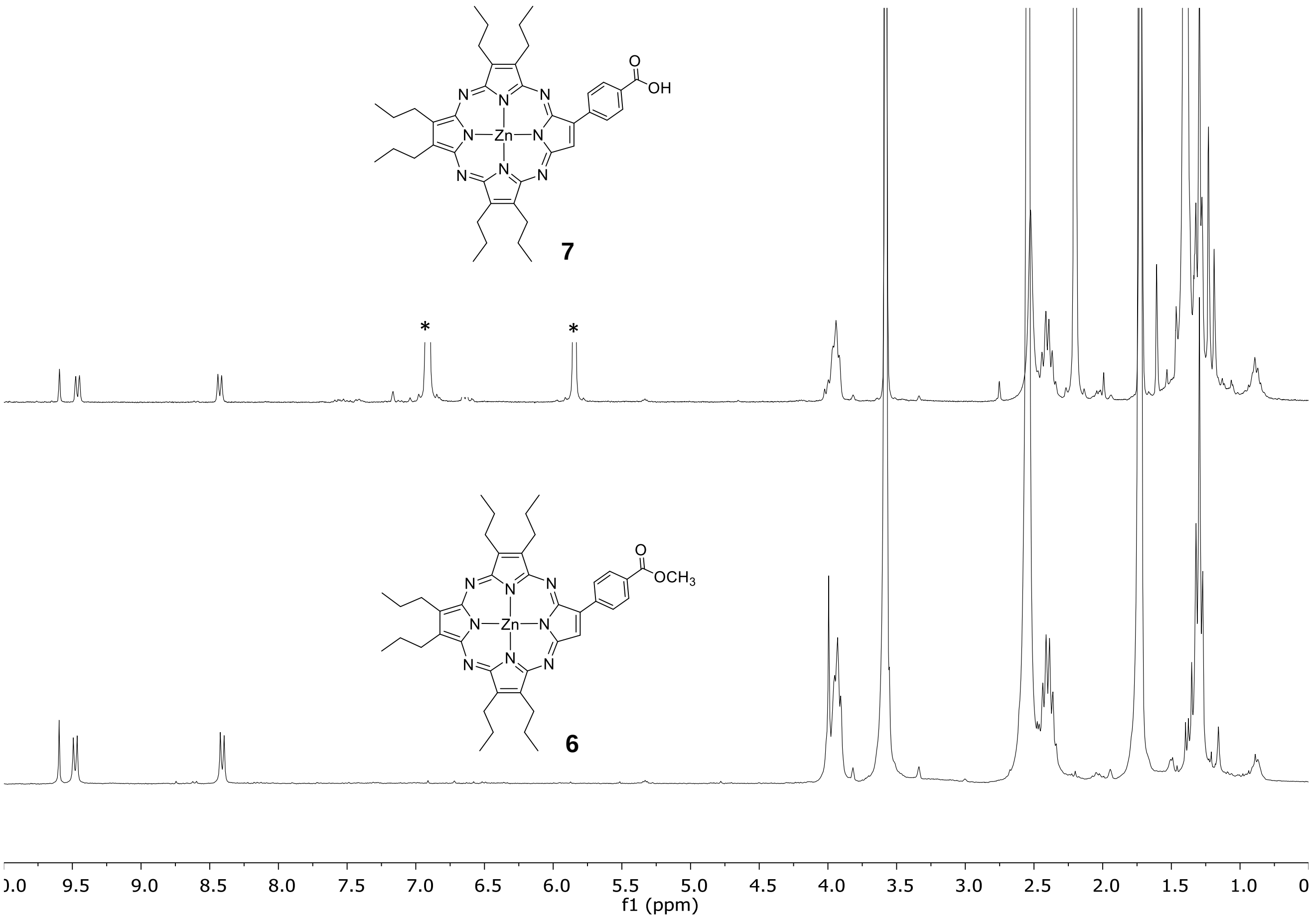

Figure S2. ^{1}H-NMR spectra of Pzs **5**, **8**, **9** and **10** in THF-d8.

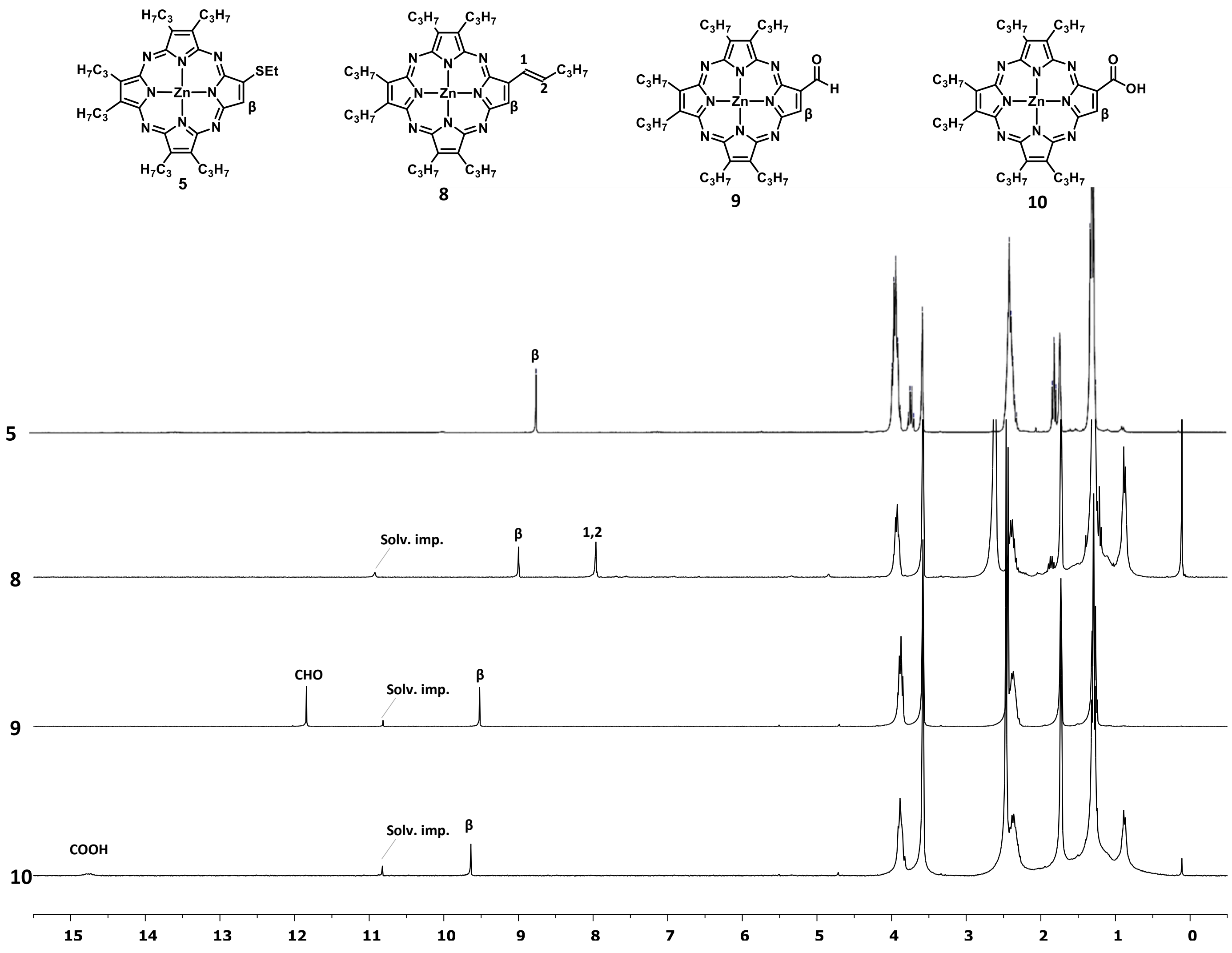

Figure S3. ^{1}H-RMN spectrum of **11** in THF-d_8 (upper) and in THF-d_8 + D_2O (lower).

PM34267 THF

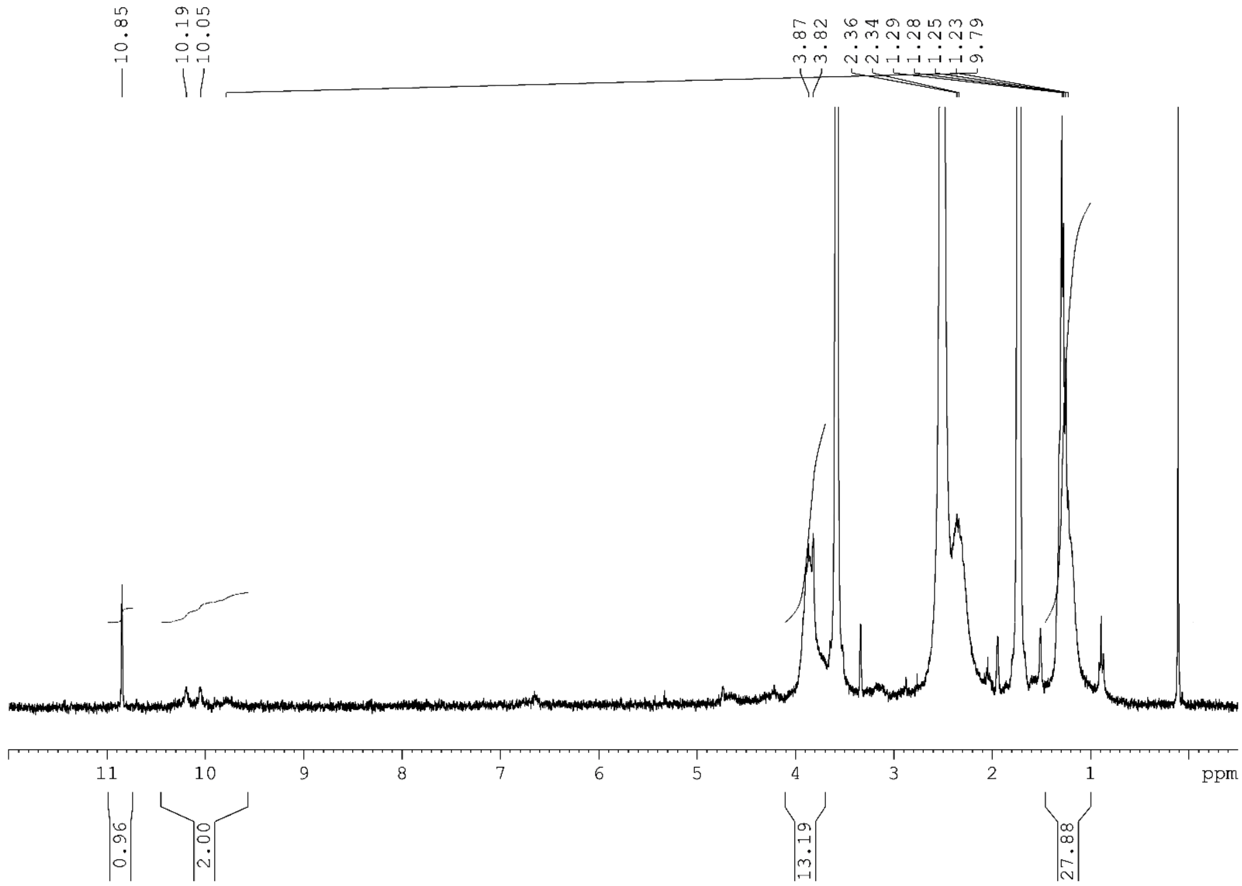

PM34267f THF + D2O

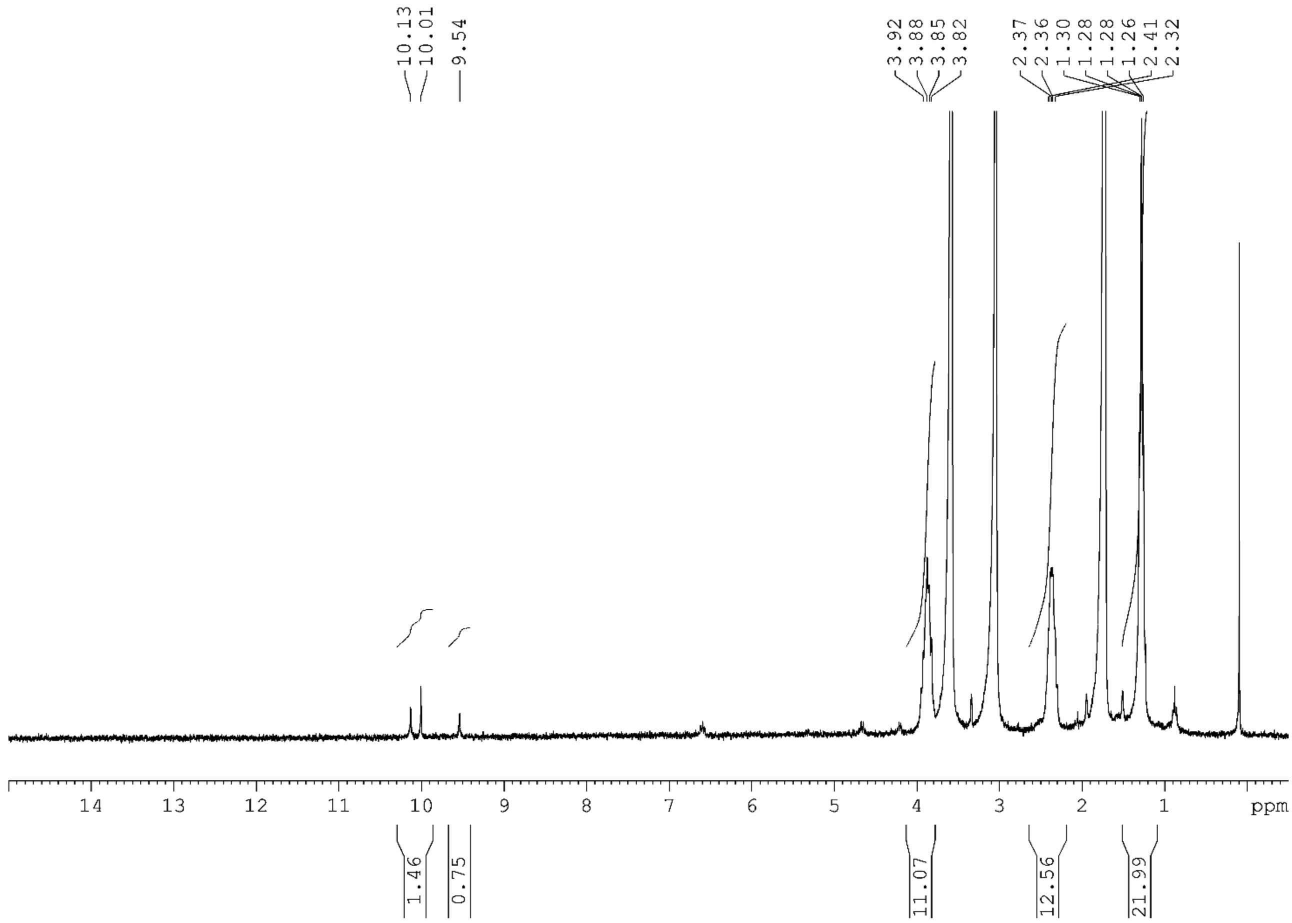

Figure S4. ^{1}H-RMN spectrum of **12** in $CDCl_3$. *H_2O

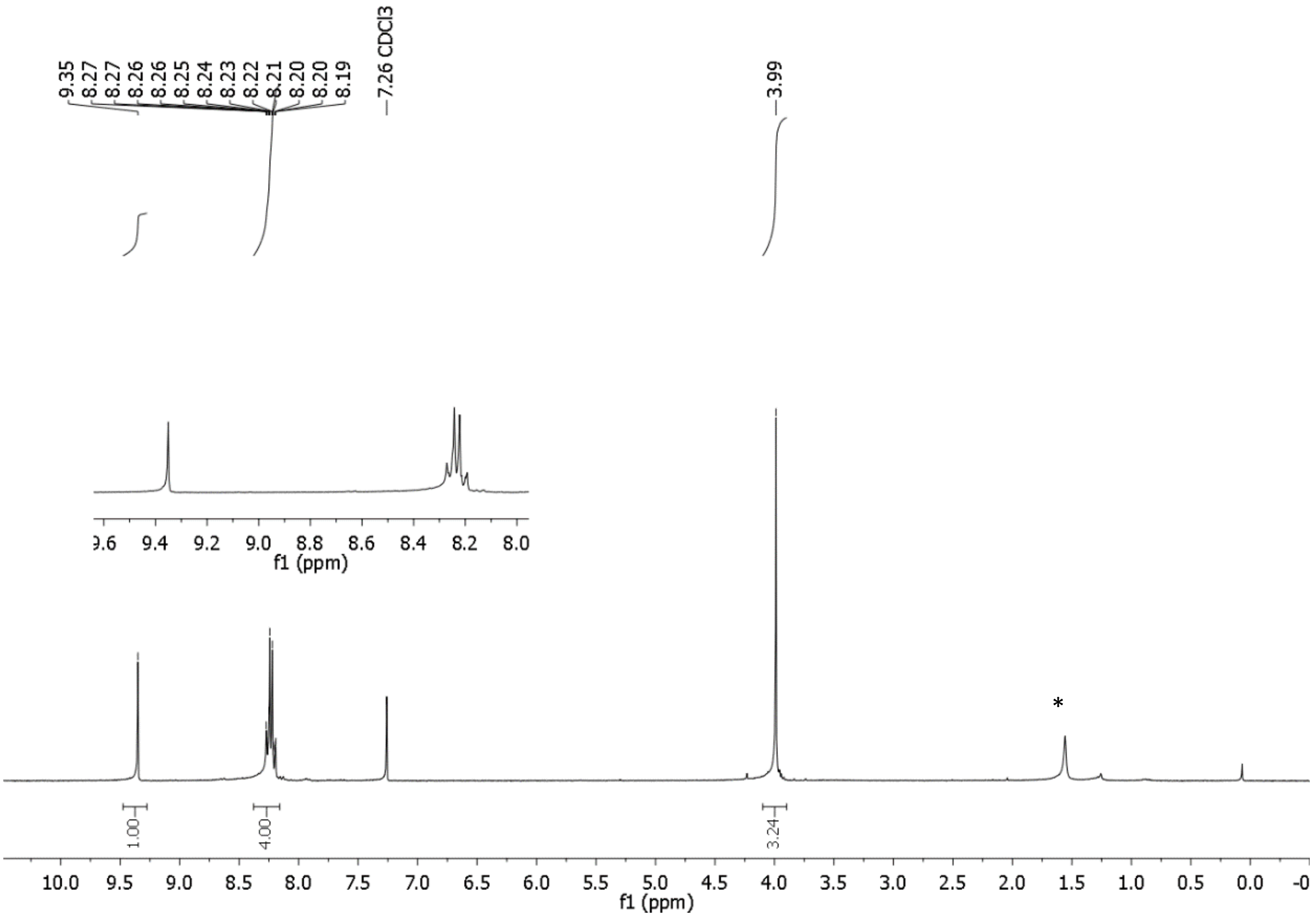

Figure S5. ^{1}H-RMN Spectrum of **13** (lower) and **14** (upper) in THF-d_8**.**

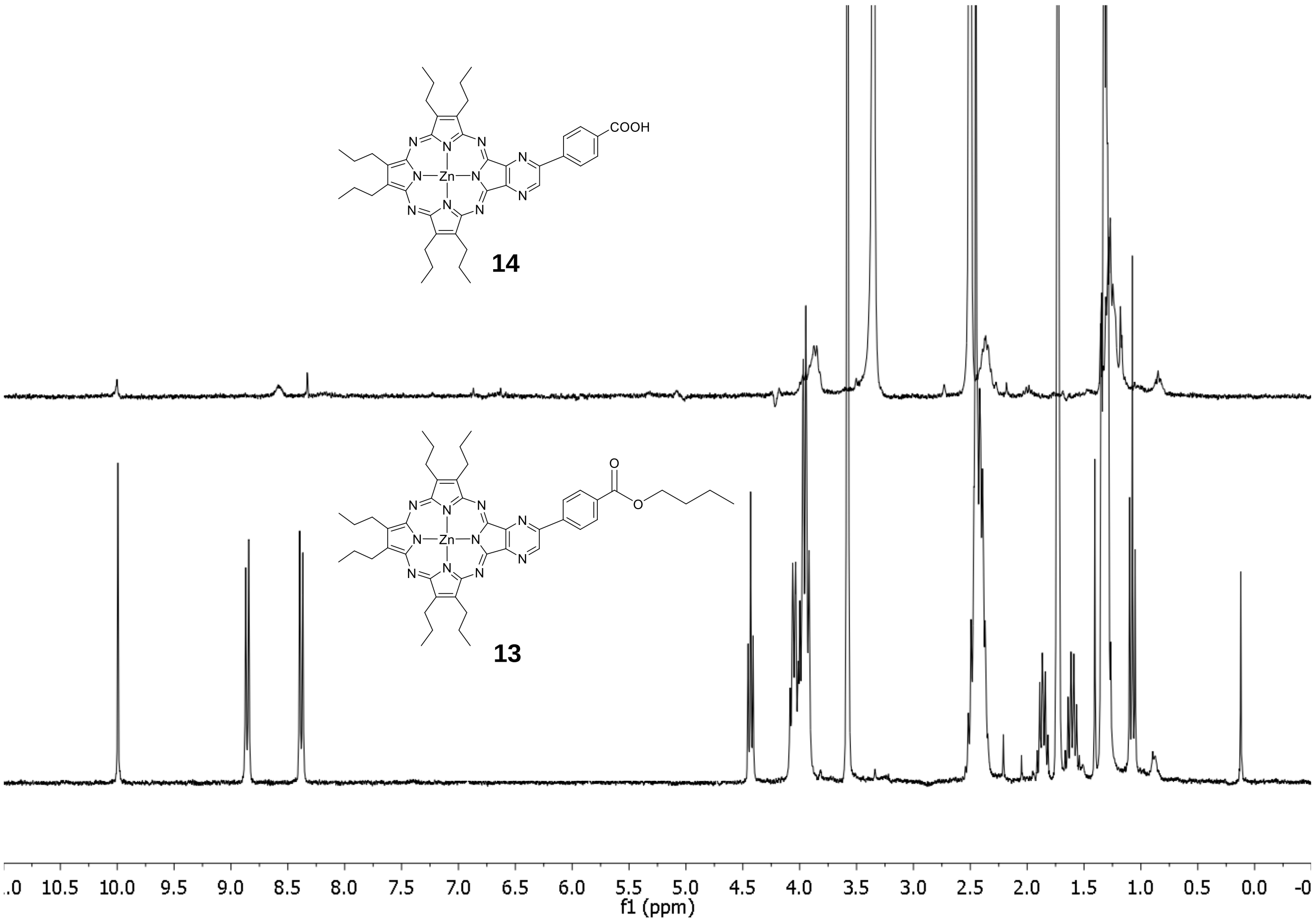

Figure S6. FT-IR spectrum (KBr) of **7**

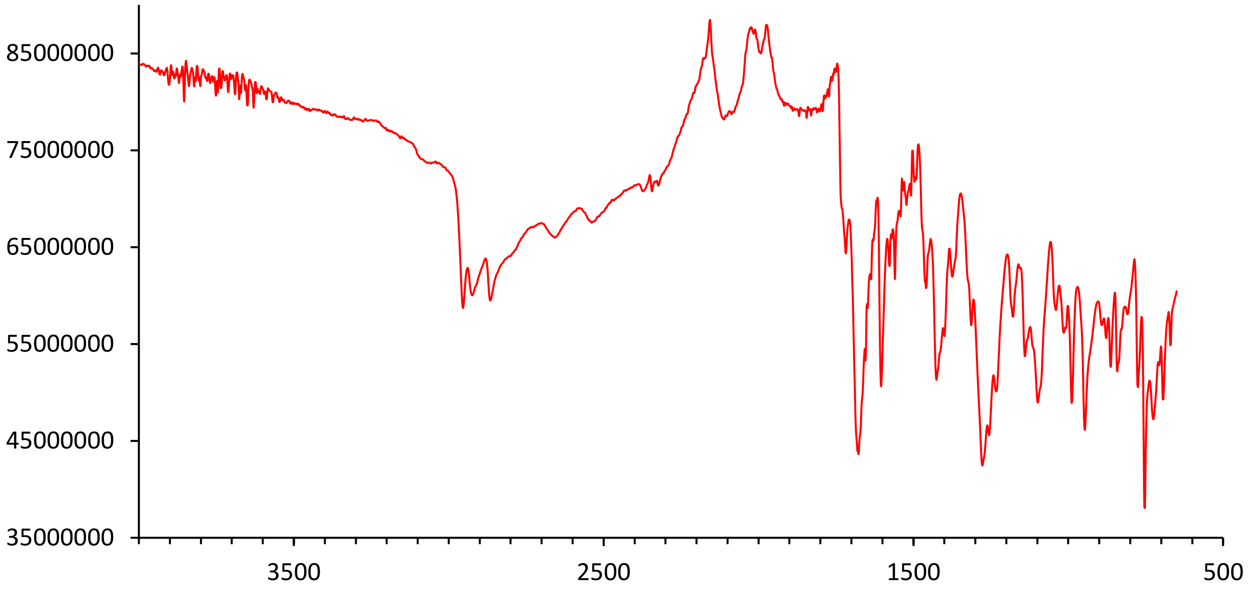

Figure S7. MS and HRMS spectra (MALDI-TOF) of **7**

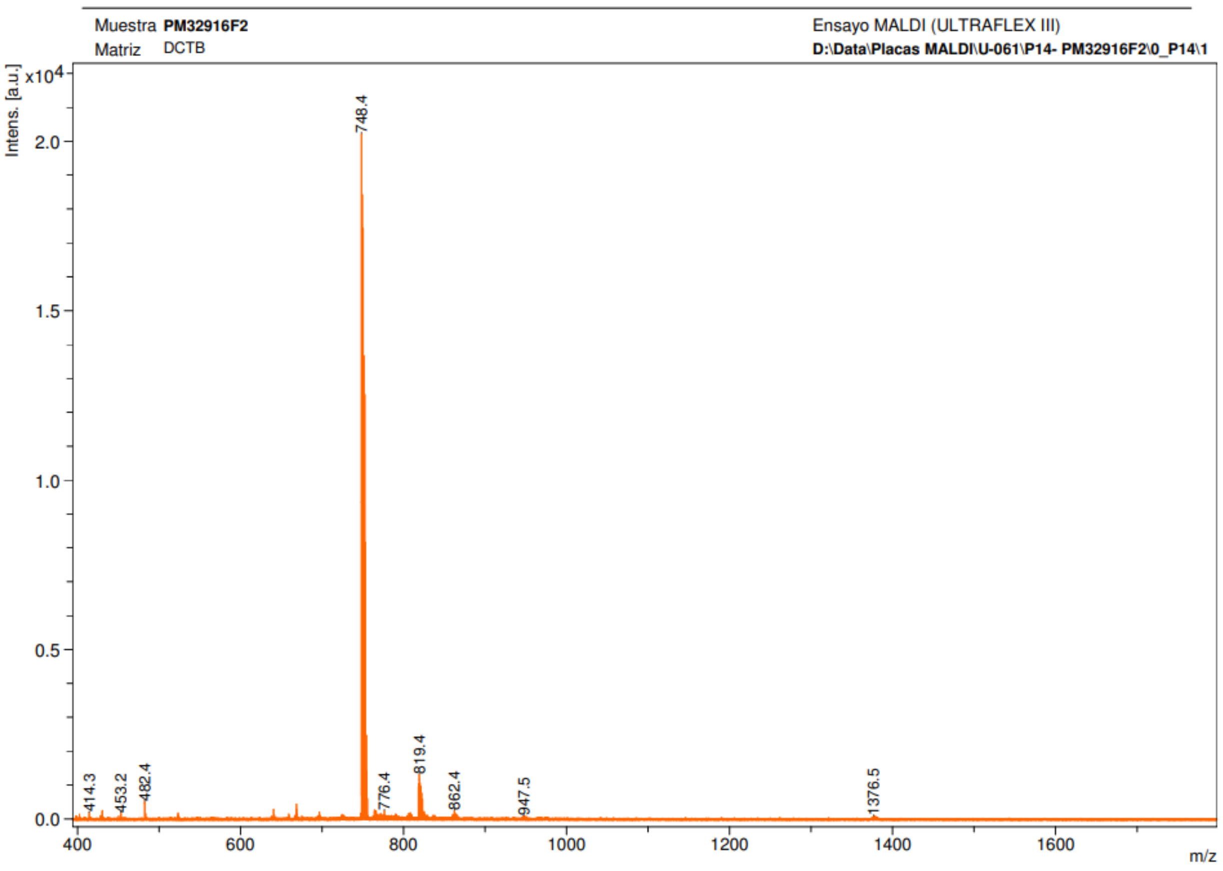

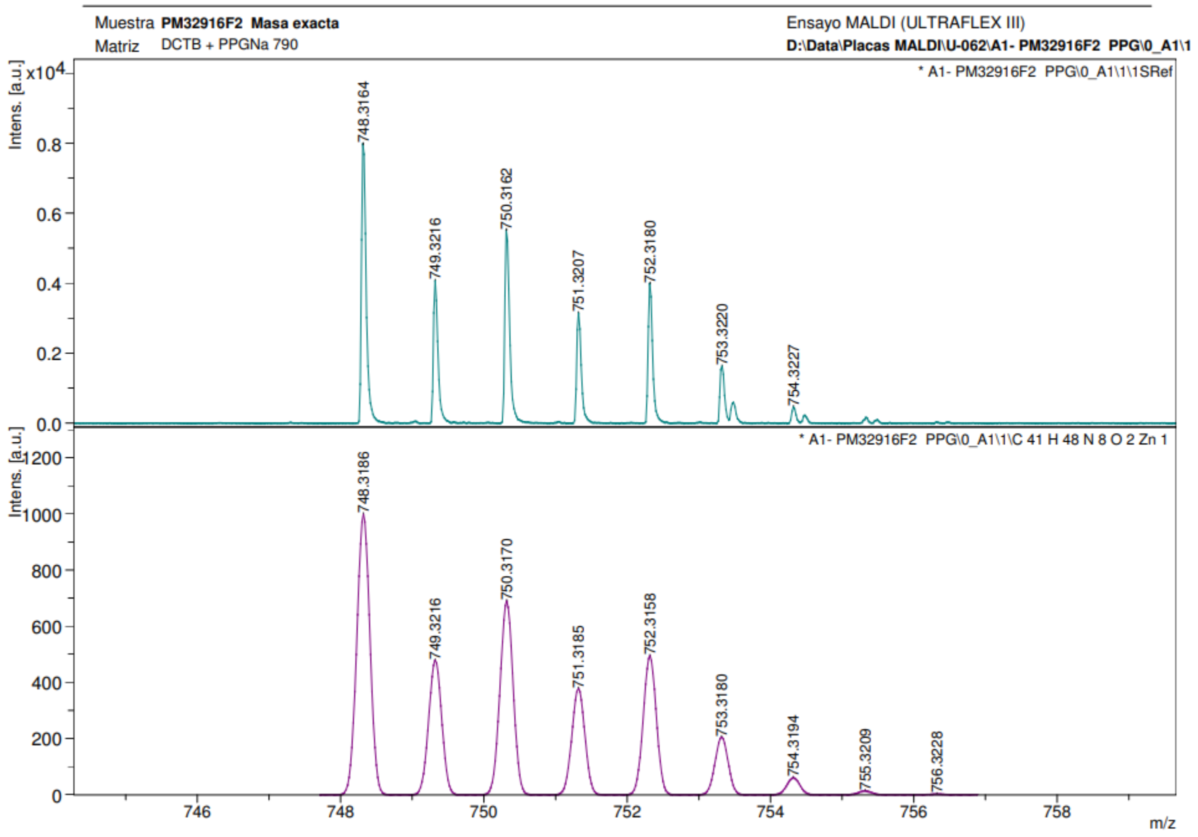

Figure S8. FT-IR spectrum (KBr) of **10**

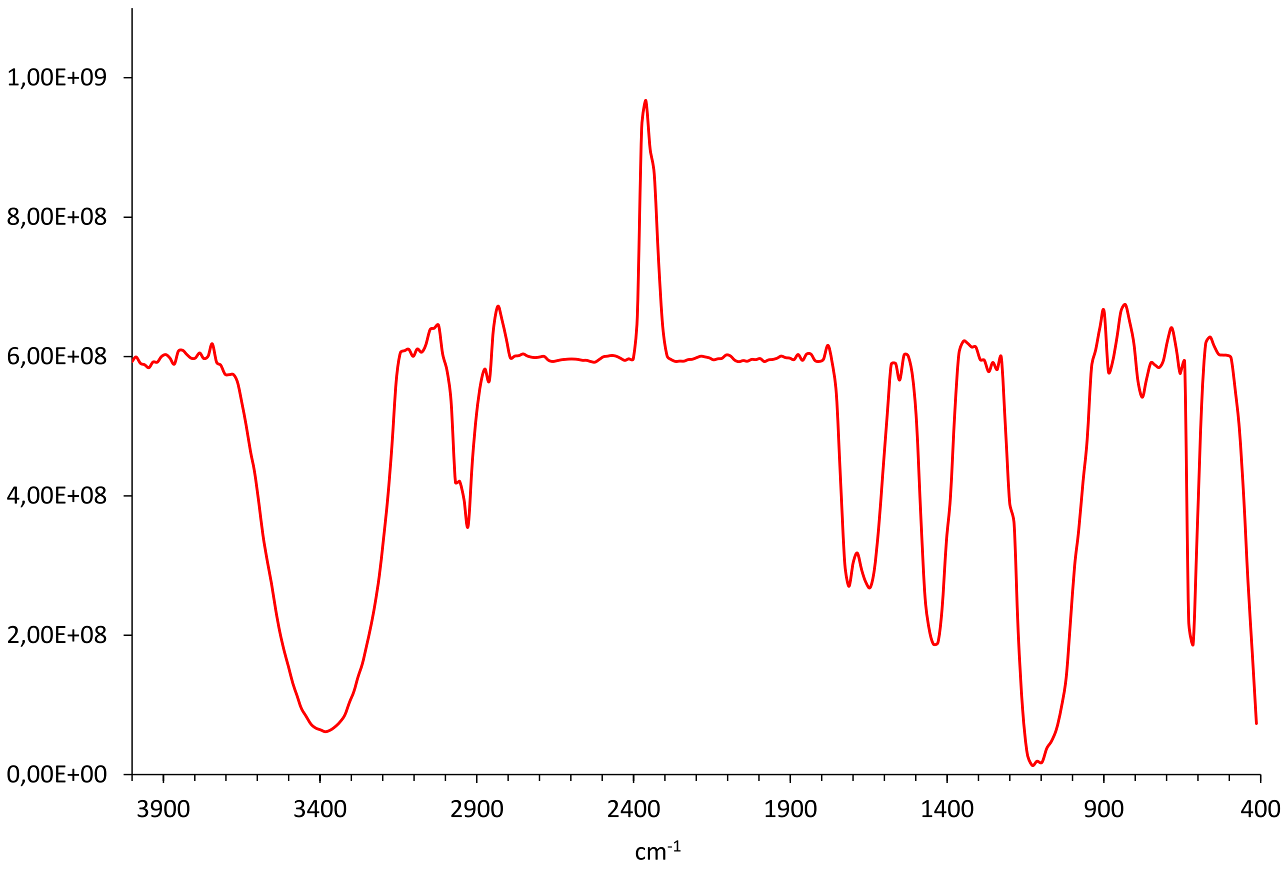

Figure S9. MS and HRMS spectra (MALDI-TOF) of **10**

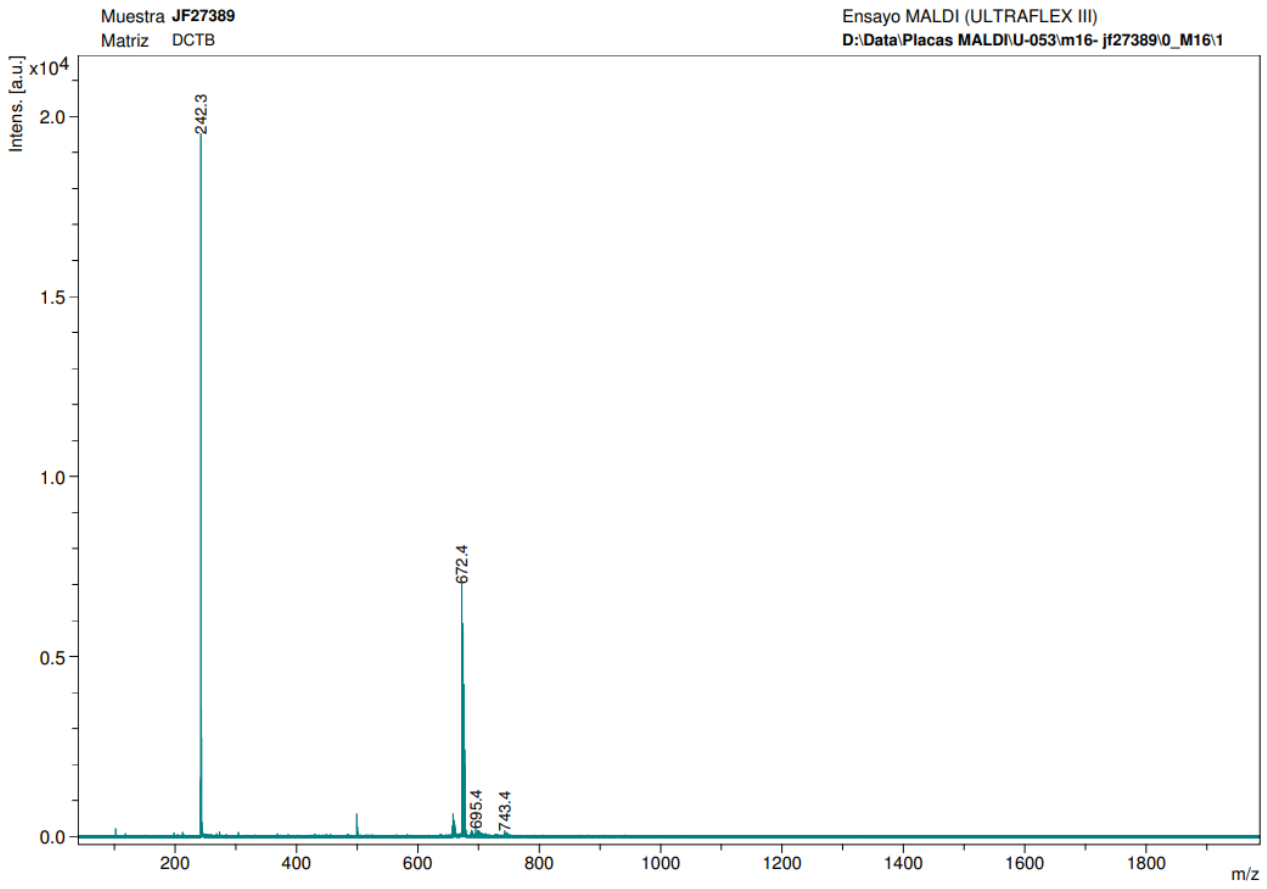

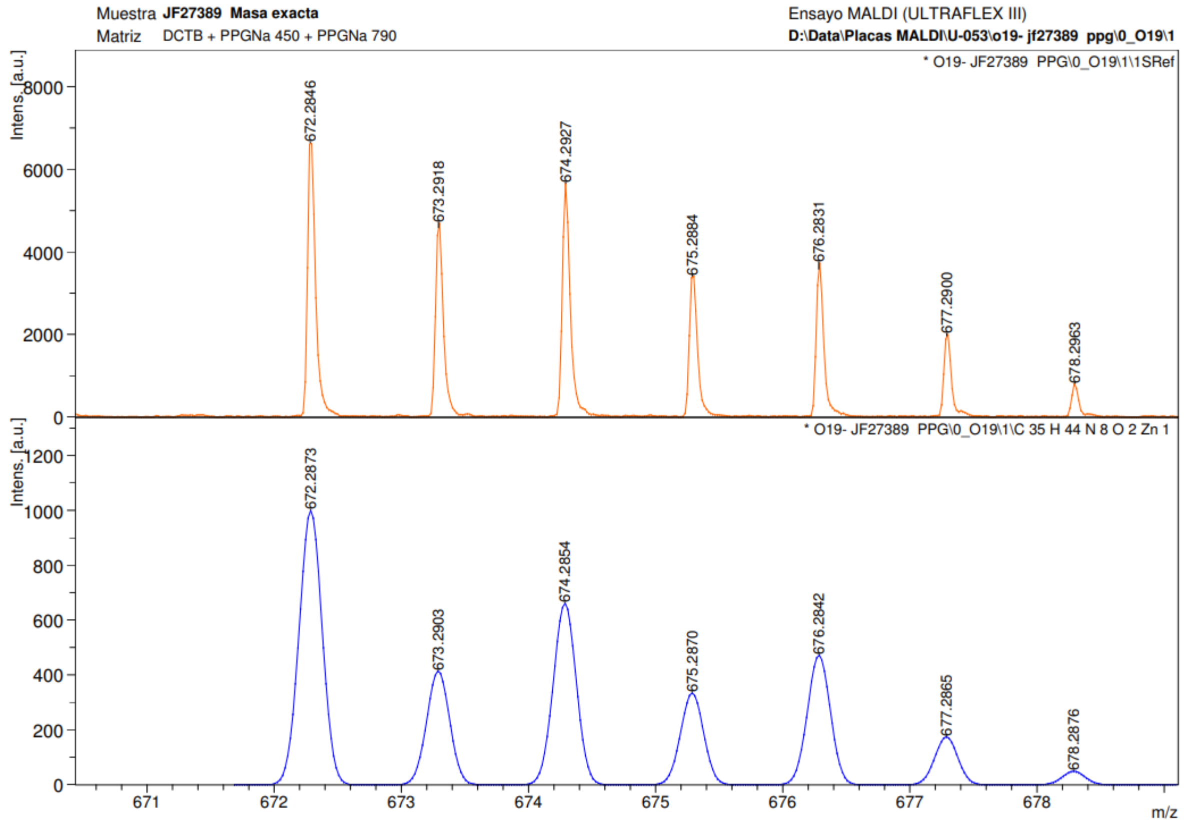

Figure S10. MS spectra (MALDI-TOF) of **11** and isotopic pattern

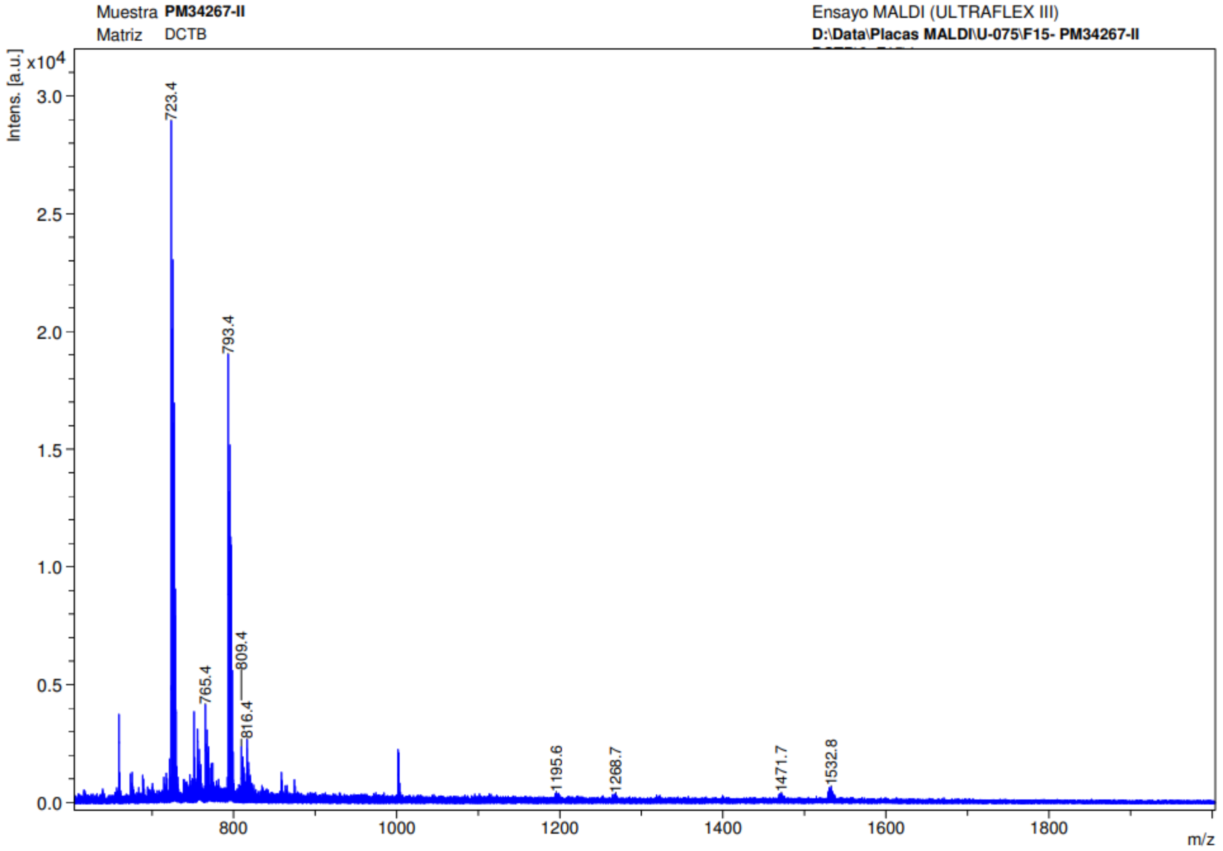

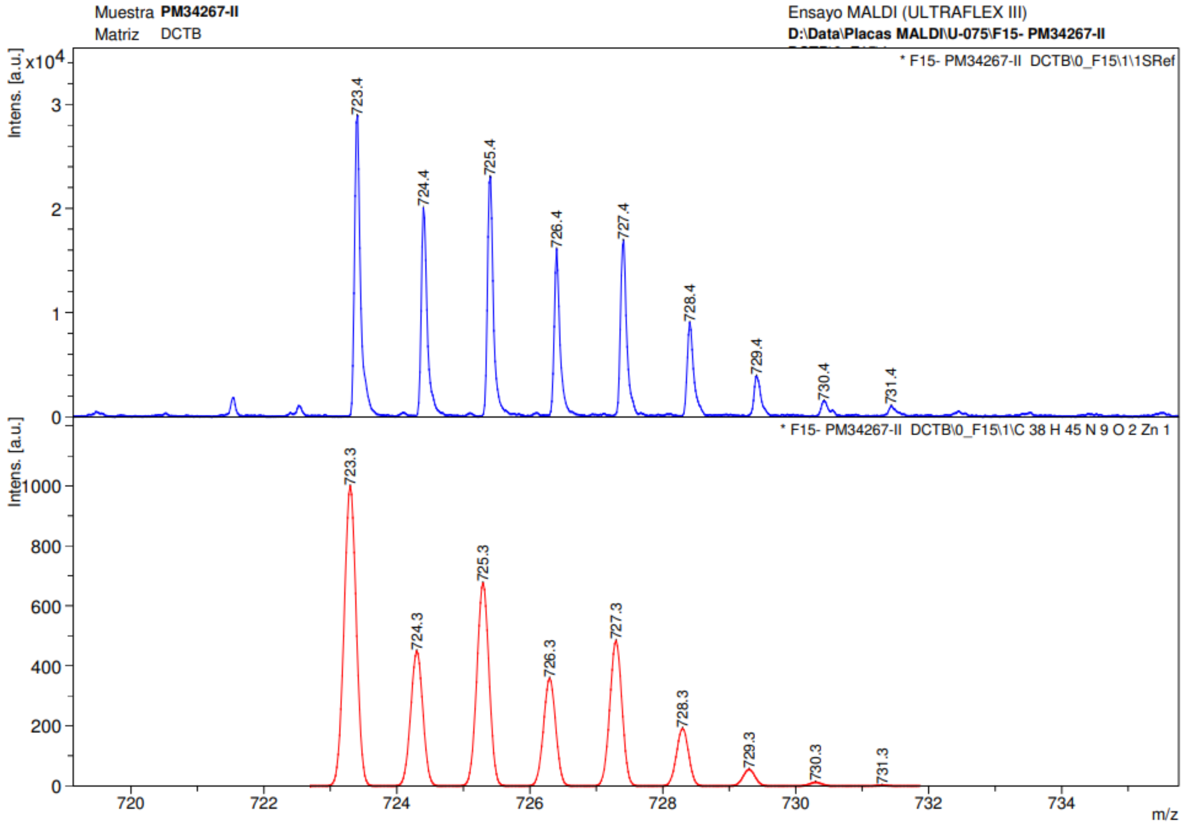

Figure S11. FT-IR spectrum (KBr) of **14**

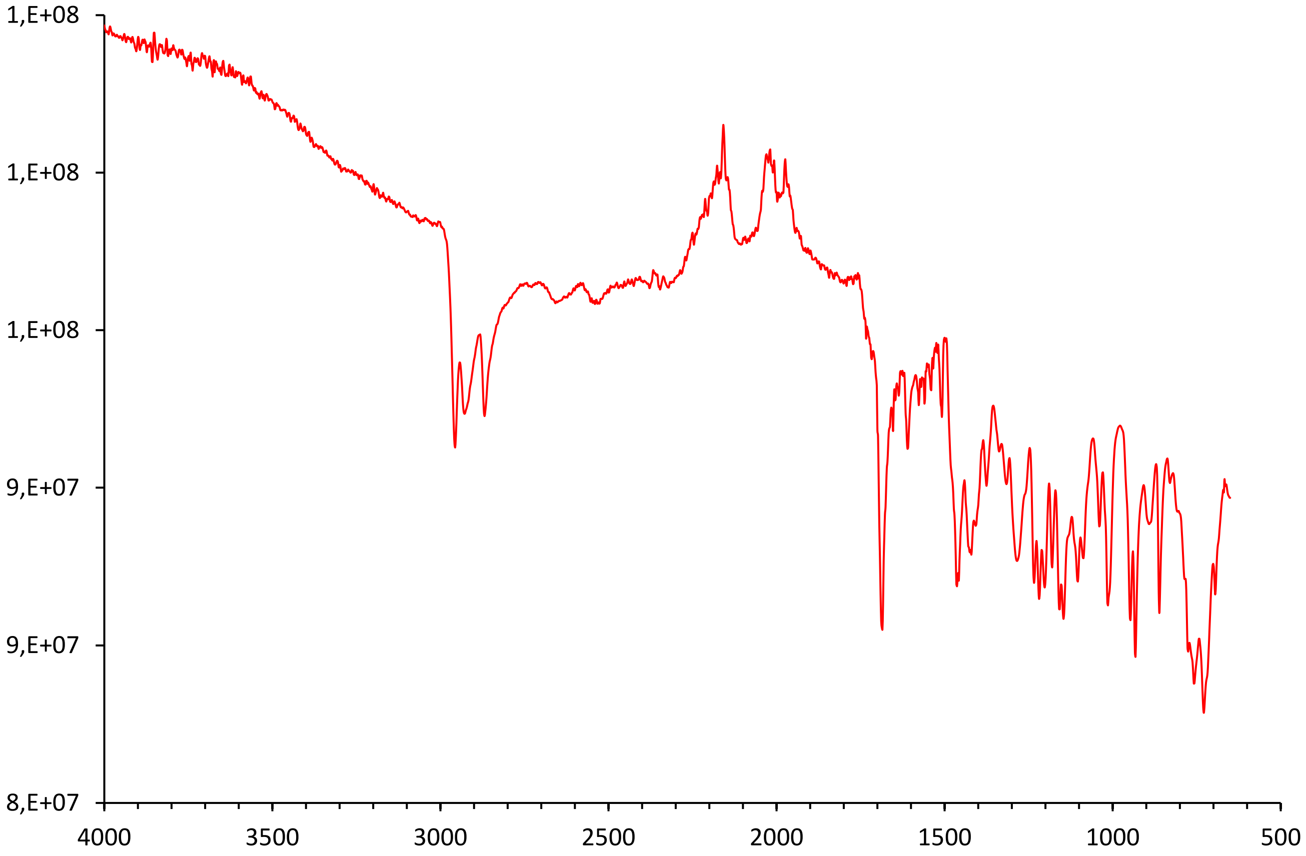

Figure S12. MS and HRMS spectra (MALDI-TOF) of **14**

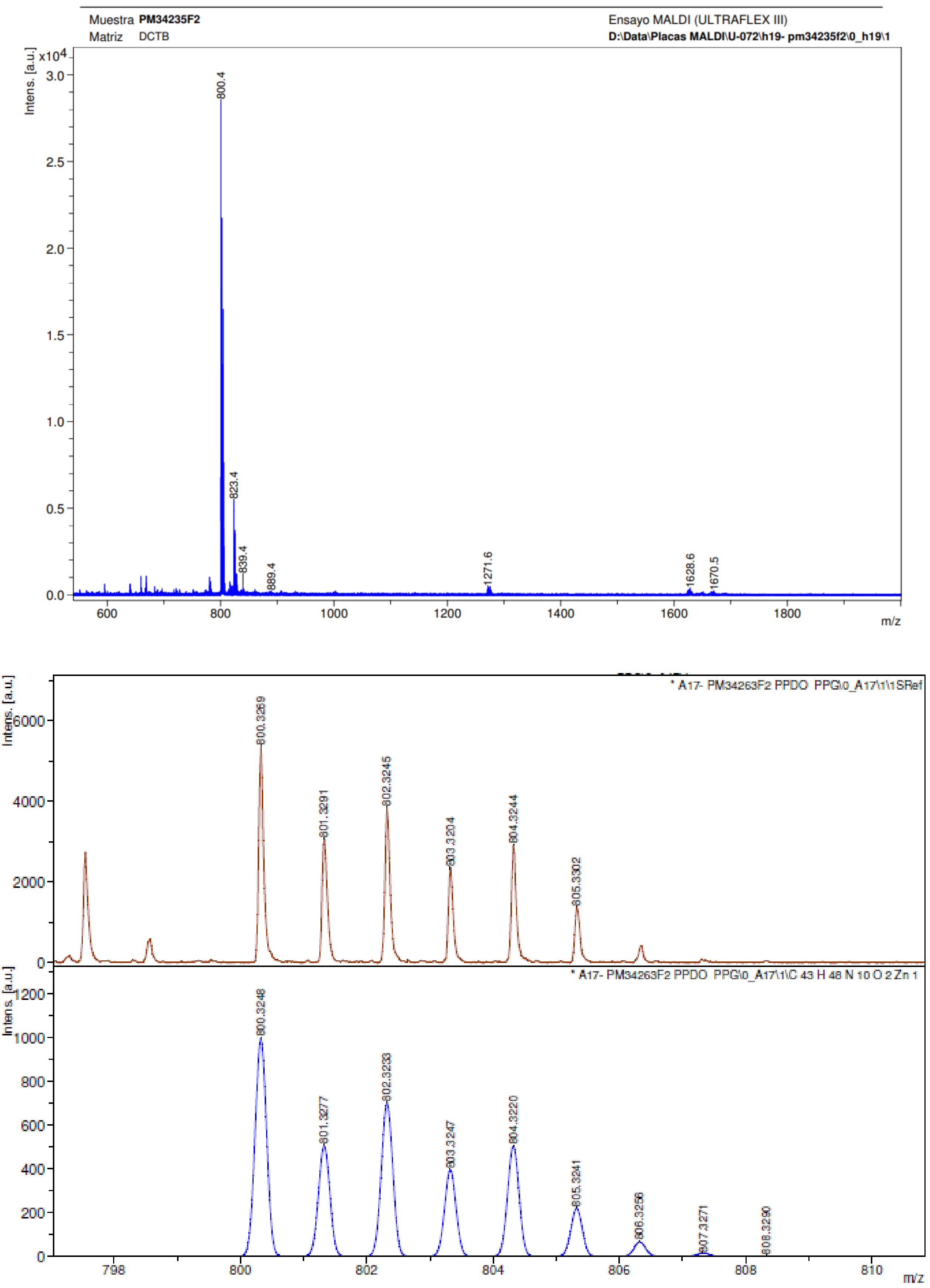

Figure S13. UV-Visible absorption spectra of Pzs **5** (light blue), **8** (green), **9** (red) and **10** (dark blue) in THF.

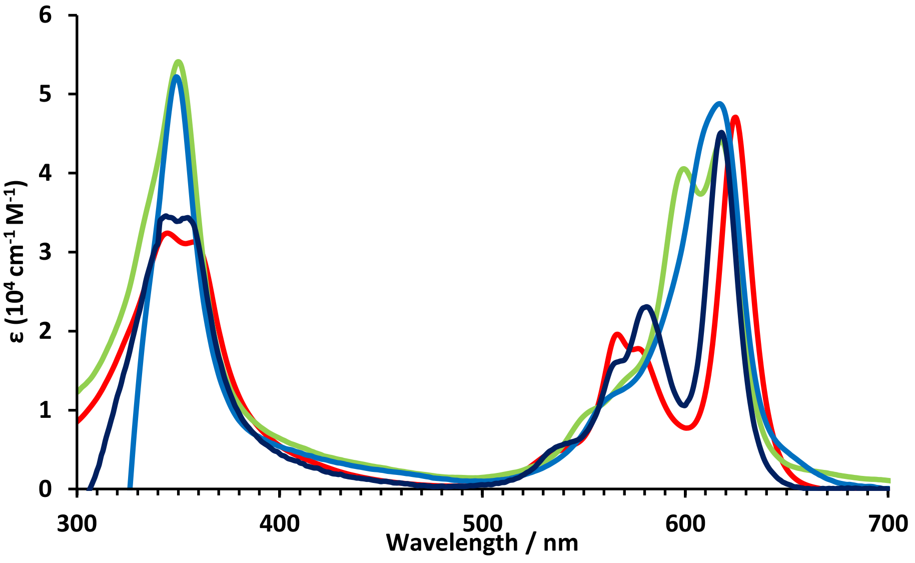

Figure S14. UV-Visible absorption spectra of Pzs **6** (light blue), **7** (orange), **11** (green), **13** (red) and **14** (dark blue) in THF

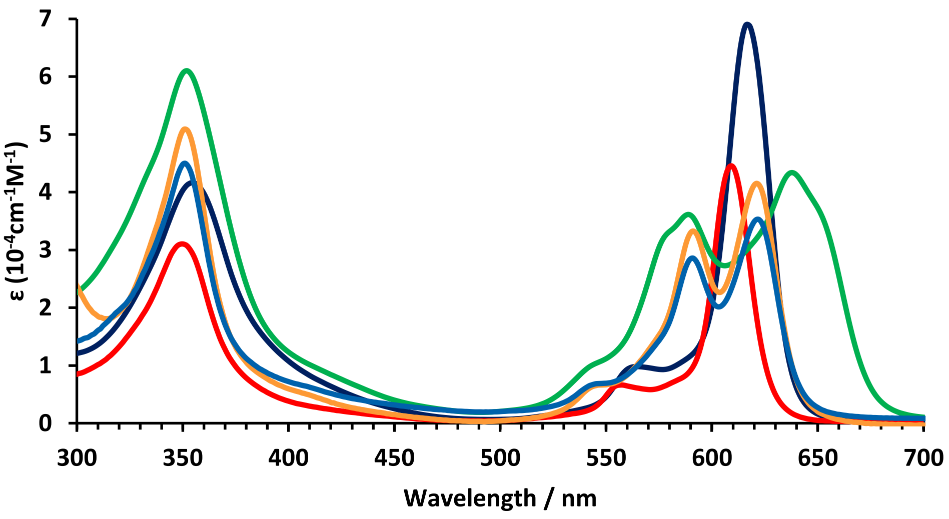

Table S1. Crystallographic data of Pz **5**.

**Formula**	$C_{37}H_{52}N_8OSZn$
**M**	722.30
**Volume/ Å^3**	1878.92(14)
**Z**	2
**Density/ g cm^{-3}**	1.277
**Crystalline system**	Triclinic
**Spatial group**	P -1
**a/ Å**	11.1803(5)
**b/ Å**	12.5793(5)
**c/ Å**	13.9769(6)
**α/ °**	81.136(2)
**β/ °**	76.267(2)
**γ/ °**	82.893(3)
**R**	0.0505

Table S2. Photovoltaic data of the DSSC devices made with **Pzs** and benchmark **TT1** adsorbed on 14 mm TiO_2 films (8.5 mm active layer + 5.5 mm of scattering layer) with an active area of 0.128 cm^2. P_{in}, incident intensity of simulated AM1.5G solar light.[a]

Dye[b]	Elect.[c] (LiI Conc.)	Cell nº	J_{SC} (mA•cm^{-2})	V_{OC} (mV)	F.F. (%)	P_{in} (mW•cm^{-2})	η (%)
**TT1+**CHENO[d]	A (0.1 M)	25	5.28	569	75.9	88.9	2.56
		26	5.89	558	74.6	88.9	2.76
**10**+CHENO[d]	A (0.1 M)	31	0.087	466	80.3	89.0	0.04
		32	0.072	445	72.2	88.7	0.03
**10**	A (0.1 M)	47	0.051	401	68.1	88.8	0.02
**7**+CHENO[d]	A (0.1 M)	33	0.826	458	74.9	88.7	0.32
		34	0.806	470	74.3	88.7	0.32
**7**+CHENO[d]	B (0.5 M)	62	1.19	403	72.7	89.1	0.39
		63	1.31	387	71.3	89.1	0.41
**14**	A (0.1 M)	27	0.424	474	70.9	88.9	0.16
		28	0.579	485	69.8	88.9	0.22
**14**+CHENO[d]	A (0.1 M)	29	0.748	480	76.1	88.9	0.31
		30	0.797	485	75.2	88.9	0.33
**14**+CHENO[d]	B (0.5 M)	60	1.13	416	73.6	89.1	0.39
		61	0.811	405	73.0	89.1	0.27
**11**+CHENO[d]	A (0.1 M)	43	0.089	400	76.4	89.0	0.03
		44	0.199	405	72.4	89.0	0.07

[a]For 1 sun irradiation, P_{in} = 100 mW cm^{-2}. [b]Dye-uptake solutions consisted of 0.1 mM of the sensitizer (with or without CHENO) in EtOH/THF (7:3) for **Pzs** or EtOH for **TT1** (dipping time 18h). [c]Iodine-based electrolytes A and B are composed of 0.6 M 1,3-dimethyl imidazolium iodine, 0.28 M 4-tertbutyl pyridine, 0.04 M I_2, 0.05 M guanidinium thiocyanate in AcCN, and differ only by the concentration of lithium iodide: 0.1M (A) and 0.5 M (B). [d]CHENO (10 mM) was incorporated in the dye-uptake solution.

Table S3. Predicted and experimental absorption bands (λ_{max}) in THF, oscillator strengths (*f*) and dominant electronic transitions, for porphyrazines **TT112**, **7**, **10**, **11** and **14**

Pz	Absorption	Predicted λ_{max} (nm)	f^a	Main contributions[b]	Experimental λ_{max} (nm)
**TT112**	B bands	334	0.83	H–7 → L (33%)<br>H–7 → L+1 (24%)<br>H–5 → L (51%)	346
	Q bands	543	0.45	H → L+1 (67%)	615
		553	0.39	H → L (68%)	
**7**	B bands	359	0.15	H–6 → L+1 (27%)<br>H → L+2 (64%)	351
		372	0.15	H–7 → L+1 (36%)<br>H–6 → L+1 (29%)<br>H–5 → L+1 (49%)	
		385	0.12	H–7 → L (53%)<br>H–6 → L (21%)<br>H–5 → L (37%)	
		389	0.13	H–7 → L (44%)<br>H–6 → L (30%)<br>H–5 → L (43%)	
	Q bands	523	0.21	H → L+1 (61%)<br>H–1 → L (27%)	591
		554	0.50	H → L (68%)	622
**10**	B bands	339	0.09	H–9 → L (64%)	344
		376	0.09	H–5 → L+1 (25%)<br>H–4 → L+1 (63%)	355
	Q bands	509	0.24	H → L+1 (64%)<br>H–5 → L (21%)	581
		539	0.36	H → L (68%)	618
**11**	B bands	366	0.52	H–7 → L+1 (28%)<br>H–6 → L+1 (44%)<br>H–5 → L (35%)<br>H → L+1 (24%)	352
		367	0.12	H–8 → L (25%)<br>H–7 → L+1 (56%)<br>H–6 → L (21%)	
		394	0.29	H–6 → L (27%)<br>H–5 → L (35%)<br>H–4 → L+1 (28%)<br>H → L+2 (43%)	
	Q bands	511	0.18	H → L+1 (63%)	589
		586	0.63	H → L (69%)	654
**14**	B bands	373	0.42	H–8 → L (21%)<br>H–8 → L+1 (21%)<br>H–6 → L (18%)<br>H–5 → L (56%)	354
	Q bands	540	0.44	H → L+1 (67%)	616
		551	0.40	H → L (67%)	

[a]Only transitions with $f \geq 0.09$ are reported.
[b]The most important contributions (> 20%) are reported.
